# Mechanochemical Synthesis and Isomerization of *N*-Substituted Indole-3-carboxaldehyde Oximes †

**DOI:** 10.3390/molecules24183347

**Published:** 2019-09-14

**Authors:** Matej Baláž, Zuzana Kudličková, Mária Vilková, Ján Imrich, Ľudmila Balážová, Nina Daneu

**Affiliations:** 1 Department of Mechanochemistry, Institute of Geotechnics, Slovak Academy of Sciences, Watsonova 45, 04001 Košice, Slovakia; 2 Department of Chemistry, Biochemistry and Biophysics, University of Veterinary Medicine and Pharmacy, Komenského 73, 04181 Košice, Slovakia; 3 NMR Laboratory, Faculty of Science, P. J. Šafárik University, Moyzesova 11, 04001 Košice, Slovakia; 4 Department of Pharmacognosy and Botany, University of Veterinary Medicine and Pharmacy, Komenského 73, 04181 Košice, Slovakia; 5 Advanced Materials Department, Jožef Stefan Institute, Jamova cesta 39, 1000 Ljubljana, Slovenia

**Keywords:** mechanochemistry, isomerization, oximation

## Abstract

Performing solution-phase oximation reactions with hydroxylamine hydrochloride (NH_2_OH·HCl) carries significant risk, especially in aqueous solutions. In the present study, four *N*-substituted indole-3-carboxaldehyde oximes were prepared from the corresponding aldehydes by solvent-free reaction with NH_2_OH·HCl and a base (NaOH or Na_2_CO_3_) using a mechanochemical approach, thus minimizing the possible risk. In all cases, the conversion to oximes was almost complete. The focus of this work is on 1-methoxyindole-3-carboxaldehyde oxime, a key intermediate in the production of indole phytoalexins with useful antimicrobial properties. Under optimized conditions, it was possible to reach almost 95% yield after 20 min of milling. Moreover, for the products containing electron-donating substituents (-CH_3_, -OCH_3_), the isomerization from the oxime *anti* to *syn* isomer under acidic conditions was discovered. For the 1-methoxy analog, the acidic isomerization of pure isomers in solution resulted in the formation of *anti* isomer, whereas the prevalence of *syn* isomer was observed in solid state. From NMR data the *syn* and *anti* structures of produced oximes were elucidated. This work shows an interesting and possibly scalable alternative to classical synthesis and underlines environmentally friendly and sustainable character of mechanochemistry.

## 1. Introduction

Aldoximes are a fundamental class of compounds employed for purification, protection, and characterization of aldehydes [1]. They are important precursors for various nitrogen-based organic compounds including amines [2], nitriles [3], and nitro compounds [4]. Typical preparation is through the solution-phase reaction of hydroxylamine hydrochloride with an aldehyde in the presence of a base [5]. However, the use of this compound in an aqueous reaction in the presence of metal ions led to a disastrous industrial accident [6]. Therefore, developing safe syntheses (including oximations) with low environmental impact are of critical importance to enabling sustainable chemical production. The solvent-free solid-state reactions for oxime synthesis are gaining popularity [7,8,9,10,11,12,13,14,15,16,17]. Among various solvent-free solid-state approaches, the addition of extra energy via microwave irradiation [18,19] or mechanical force by grinding (mechanochemistry) [20,21,22,23], are popular research topics. This energy input accelerates reactions and can also lead to altered selectivity [24]. Research on mechanochemical synthesis in general is increasing in frequency as it represents an environmentally friendly and “green” method of synthesis. In recent years, significant advances have been achieved in both inorganic and organic mechanochemistry [25,26,27,28,29,30,31,32,33,34,35]. A wide array of organic reactions has been implemented mechanochemically with good yields [24,36,37,38,39]. This is also the case of the transformation of aldehydes into aldoximes [22,23].

**Figure 1:**
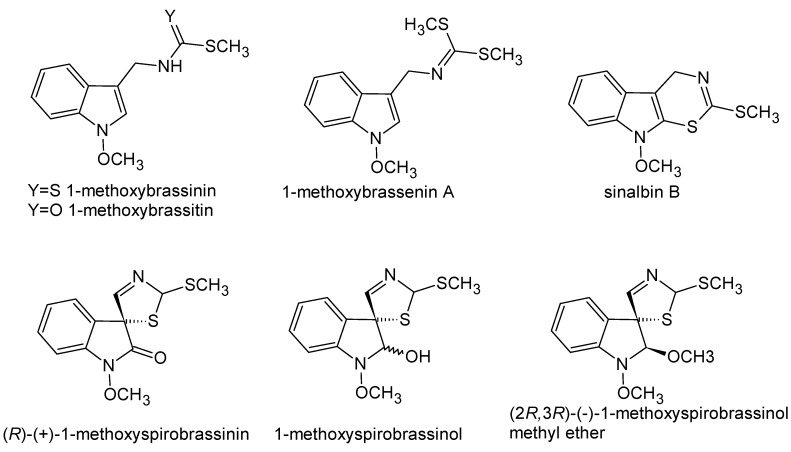
Structures of indole phytoalexins.

Although a broad range of aldehydes have been used in previous reports on the solid-state synthesis of oximes [22,23,40,41], the reactions of *N*-substituted indole-3-carboxaldehydes, are unexplored. Regarding 1-methoxyindole-3-carboxaldehyde, Somei’s method starting from indoline is typically used for its synthesis [42,43,44]. One reason for our interest is that 1-methoxyindole-3-carboxaldehyde oxime is a reaction intermediate in the long synthetic pathway to cruciferous phytoalexins that exhibit antimicrobial and antiproliferative activity (the structures of some phytoalexins are provided in Figure 1) [45,46,47,48].

There are many reports on the solution-based syntheses of *N*-substituted indole-3-carboxaldehyde oximes from their aldehydes [49,50,51,52,53,54]. Regarding the 1-methoxy analog, Hanley et al. firstly reported the conversion of the corresponding aldehyde to oxime with the 50% yield [55]. The authors did not investigate the isomerization of *syn* and *anti* oximes and merely used the reaction mixture for further syntheses. In the same year, Kawasaki et al. performed the same reaction in pyridine with 98% yield [56]. The individual *syn* and *anti* isomers were separated by column chromatography and the possibility of mutual isomerization was shown. Later on, the same reaction was also performed by Pedras et al. with quantitative yield [57]. 

Here, we report the simple solvent-free solid-state syntheses of four *N*-substituted indole-3-carboxaldehyde oximes. Different ratios of isomers were found depending on reaction conditions. The isomerization of crude reaction mixtures, as well as that of pure isomers was also investigated showing different outcome depending on whether it was prepared by a solid-state route or in solution. We demonstrate the simplification of one reaction step in the synthetic pathway to indole phytoalexins [47,48] and their analogs [58,59].

## 2. Results and Discussion

### 2.1. Mechanochemical Synthesis of 1-Methoxyindole-3-carboxaldehyde Oxime (Syn, Anti-***1***)

**Scheme 1:**
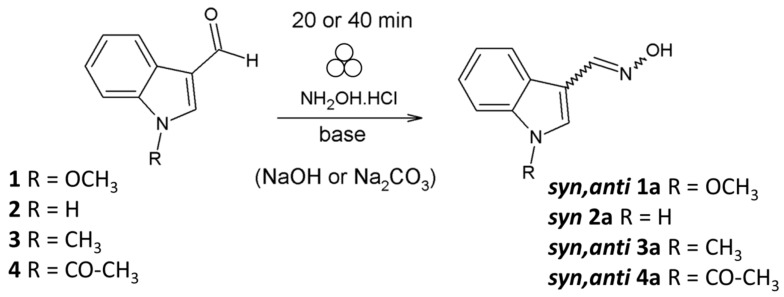
The general reaction scheme for the synthesis of *N*-substituted indole-3-carboxaldehyde oximes from the corresponding aldehydes.

In the solution-phase syntheses of the 1-methoxyindole-3-carboxaldehyde aldoxime, sodium carbonate [55], or pyridine [56] was used as a base. However, solid-state approaches have employed sodium hydroxide for the substituted benzaldehydes aldoxime production [23]. In this study, the versatile approach reported by Aakeroy et al. was used as a starting point for our system. However, we found it is necessary to use high-energy ball milling to obtain acceptable yields. The general reaction scheme, also for the non-substituted and other *N*-substituted analogs for which the results will be reported in Section 2.4, is shown in Scheme 1.

Initially, attempts to reproduce the procedure reported in [23] yielded unsatisfactory results, most likely due to the electron-donating resonance effect of indole nitrogen which decreases the electrophilicity of carbonyl carbon [60]. More details regarding this experiment are provided in the ESI (Table S1) Optimization of the mechanochemical reaction under study was carried out through an extensive variation of the reaction parameters (Table 1). Since excess hydroxylamine hydrochloride has been used in the solution-based report on this reaction [55], we hypothesized that an increase in the amount of hydroxylamine and hydroxide would lead to improved yields. In this optimization, up to 5.2 equivalents of NH_2_OH·HCl and 2.5 equivalents of base were used (Table 1, entries 1–2). In both cases, the reaction went to completion according to NMR data. However, the reaction with 3.1 equivalents of hydroxylamine (Table 1, entry 1) exhibited slower kinetics (it was finished after 30 min) and no product isomerization. Therefore, in the next experiment (Table 1, entry 3), 5 equivalents of hydroxylamine were maintained, but an effort to further reduce amount of NaOH (to 2 eq) was made. As seen in the table, complete conversion was again achieved. In order to investigate the effect of altering the amount of hydroxide on the isomerization process, the same experiments under neutral conditions (5 equivalents of NH_2_OH·HCl and 5 equivalents of NaOH was realized (Table 1, entry 4)). The reaction went to completion also in this case, just the isomers ratio was significantly different, but this will be discussed later.

Unfortunately, the reaction mixtures extracted from tungsten carbide milling vessel were significantly contaminated by particles coming from the milling media and chamber walls. The effect of wear in the field of organic mechanochemistry has been studied in the past [61,62,63,64,65,66] and it has been proposed that agate is the most suitable material from the contamination point of view [67]. Based on this, it was hypothesized that performing the reaction in an agate milling vessel would produce similar results with less wear. Moreover, it is known that liquid assisted grinding (LAG), the addition of substoichiometric amounts of solvent to a milling reaction, can significantly reduce the time of milling (thus also wear) and potentially also the stereochemical distribution of the reaction products [38,68,69,70]. Therefore the next experiment was performed in an agate vessel using LAG (Table 1, entry 5). Reaction completion was achieved after 30 min of milling with no observable contamination. The neat reaction (Table 1, entry 6) took slightly longer time (40 min), but the ratio of isomers was the same as when using LAG. Based on these results LAG was not used in the further experiments. However, the η value was just 0.022 μg/mL, which means that the process was almost neat. The influence of LAG on this process both in terms of solvent amount and type could be a topic for separate study in future.

Although the initial results with the agate milling material were positive, it was difficult to extract the material from the milling vessel. Despite exhaustive mechanical efforts, only half of the reaction mass could be mechanically extracted from the milling vessel. This is reflected in a low isolated yield (Table 1, entry 5). Agate is prone to fracture and the reaction mixture was most probably lost in the cracks in the vessel walls. However, an ethyl acetate extraction of the whole milling vessel facilitated recovery of the reaction products (Table 1, entry 6).

For comparison, an experiment with sodium carbonate, a weaker base than sodium hydroxide, but being less hygroscopic and easier to handle, was completed (Table 1, entry 7). As can be seen, a good yield was obtained upon switching bases. The amount of base and hydroxylamine was slightly higher (2.7 and 5.2 eq, respectively), in order to simulate the conditions in [55]. The reaction was completed in 40 min, so the necessary reaction time was longer in comparison with experiments implementing NaOH.

In Figure S1, the ^1^H-NMR spectra of the purified reaction mixtures are provided, which show no residual aldehyde for entries 1–7. The *syn*:*anti* oxime ratios presented in the Table 1 will be discussed in detail in the following section. It follows from all these considerations that the ideal experimental conditions are the ones applied for entry 3 in Table 1 (marked in bold). This experiment is also the most favorable from the *syn*:*anti* oxime ratio point of view.

Additionally, events leading to increase in gas pressure in the chamber were detected during the reaction using the special jar equipped with a pressure sensor. A detailed description of these phenomena is provided in the ESI (Figure S2 and the description therein).

In general, the samples after ball milling were significantly contaminated with particles of milling media. This was noted in the increased crude reaction mixture mass and color change (white to black). Purification was achieved using a flash chromatography on silica gel and isolated yields are reported in Table 1. The lowest yields (around 20%) were due to mechanical losses in extracting the product from the agate milling vessel (Table 1, entry 5). The isolated yield from the tungsten carbide milling vessel varies from 45% for the experiment with Na_2_CO_3_ to values above 75% for the experiments with NaOH. When a small amount of ethyl acetate was used to extract the powder from the vessel (Table 1, entries 3, 4, and 6), the isolated yields could be increased above 80% and 94% for the agate and tungsten carbide milling vessels, respectively. 

In order to compare the reactions conducted mechanically with standard synthetic approaches, three reactions were performed in solution using the hydroxylamine and base ratios optimized for the mechanochemical synthesis (Table S1, entries S1–S3). In all cases, the reactions were completed within 1 h. However, the ratio of isomers was different from the experiments utilizing ball milling, as will be discussed later.

### 2.2. Aging Experiments for the Synthesis of 1-Methoxyindole-3-carboxaldehyde Oxime (Syn, Anti-***1a***)

In order to investigate whether the reaction can reach completion simply by a short activation via milling followed by storage of the reaction mixture (aging), milling of the reagents was performed for 5 min in the agate vessel (Table 1, entry 8) and for 15 s in the WC vessel (Table 1, entry 9). Longer agate milling times were used due to the lower density of silica versus tungsten carbide (2.65 g/cm^3^ versus 15.63 g/cm^3^).

The reaction progress can be easily followed by ^1^H-NMR spectroscopy (Figure S3), as the chemical shifts of the protons of starting aldehyde (CHO; 9.88 ppm) are significantly different from the ones for *syn* (H-8; 7.83 ppm) and *anti* (H-8; 8.21 ppm) oximes. In the agate experiment (Table 1, entry 8), the starting aldehyde was almost completely consumed already after 1 day. The transformation of aldehyde in the experiment using WC grinding (Table 1, entry 9) proceeded much more slowly as a significant amount of the aldehyde was still present after 5 days. The *anti-syn* oxime isomerization was observed in both systems and it will be described in detail later. Since it was found that the reaction proceeds to some extent also in DMSO (Table S2), the NMR measurements were performed immediately after dissolution of the reaction mixture. After washing, the final samples still contained 3% and 15% (respectively) of unreacted aldehyde (Table 1, entries 8 and 9). It was concluded, that the surface energy of the reagents, which was increased by short milling through the increase of surface area and generation of defects, was insufficient for the completion of the reaction. However, these results suggest that there may be an optimal processing time that would result in a complete reaction.

### 2.3. Isomerization of 1-Methoxyindole-3-carboxaldehyde Oxime (Syn, Anti-***1a***)

It is well-known that oximes can be present as *syn* and *anti* (*Z* and *E*) isomers and they can undergo isomerization [71]. In the case of 1-methoxyindole-3-carboxaldehyde oxime, the ratio of the isomers was not reported. To obtain these data, we performed an experiment according to the literature [55] and the obtained ratio of *syn*:*anti* oxime isomers was 40:60 (Table S1, last entry). In this work, we turned our attention to the possible mutual isomerization of oxime isomers. It can be seen from Table 1 showing the results for the solvent-free synthesis that the *syn*:*anti* ratios were different, depending on the experimental conditions used, however, in most reactions, the final content of *syn* isomer was significantly higher, so the isomerization process must have taken place. In the solution-based reactions performed for comparison, the prevalence of *syn* isomer was also evident, being higher when the reaction mixture was left to isomerize (Table S1, entry S2). However, if a 1:1 hydroxylamine:hydroxide ratio was used, the *anti* isomer was favored despite longer storage (Table S1, entry S3).

In the case of all solvent-free reactions performed with NaOH, there was an excess of the *anti* isomer immediately after the reaction (according to TLC). However, when the residual non-reacted hydroxylamine was not removed from the reaction mixture, the transformation from *anti* to *syn* isomer occurred, and after few days, the *anti* isomer was nearly entirely converted to the *syn* one for the experiments where a significant excess of the equivalents of NH_2_OH·HCl over NaOH was used (Table 1, entries 2, 3, 5, 6, 8 and 9). This was discovered by performing time-resolved TLC of a selected experiment. Details can be found in the ESI (Figures S4 and S5).

**Figure 2:**
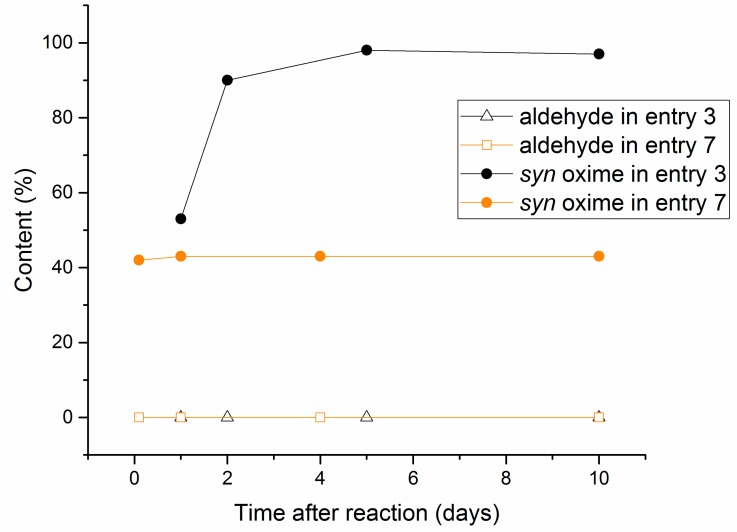
Amounts of non-reacted aldehyde **1** and *syn* oxime **1a** in selected experiments performed with a Pulverisette 7 Premium Line mill in a WC jar determined from ^1^H-NMR spectra. Time of milling for entry 3: 20 min and for entry 7: 40 min. In entry 3, 5 eq. NH_2_OH·HCl and 2 eq. NaOH were used. In entry 7, 5.2 eq. NH_2_OH·HCl and 2.7 eq. Na_2_CO_3_ were used. The data reported after 10 days are for the washed samples.

**Figure 3:**
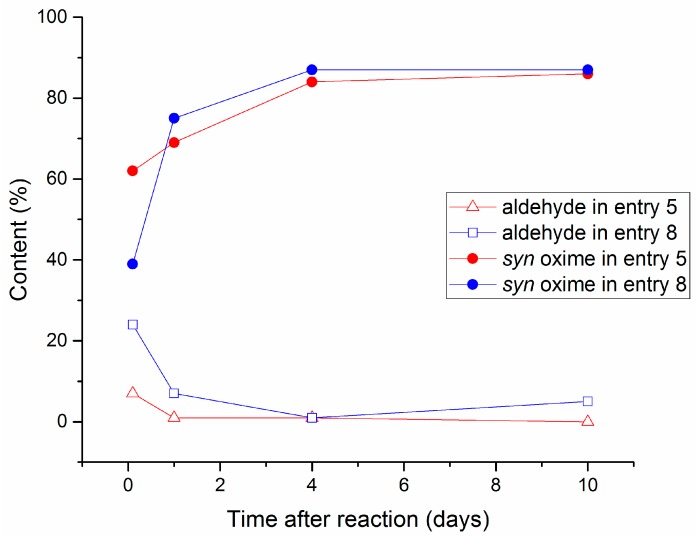
Amounts of non-reacted aldehyde **1** and *syn* oxime **1a** in selected experiments performed in Pulverisette 6 mill in agate chamber determined from ^1^H-NMR spectra. Time of milling for entry 5: 30 min, LAG and for entry 8: 5 min. In both entries, 5 eq. NH_2_OH·HCl and 2 eq. NaOH were used. The data reported after 10 days are for the washed samples.

This process can also take place when the reaction has not gone to completion. The two phenomena, namely the completion of the studied reaction and the isomerization of the products can coincide. By means of ^1^H-NMR spectroscopy, we followed these processes in the selected experiments (Figure 2 and Figure 3).

In Figure 2, the influence of base type can be seen. The reaction went to completion during the milling process in both cases (no aldehyde was detected). The residual hydroxylamine brought about almost complete transformation of the *anti* isomer into the *syn* when NaOH was used (ideal experiment, Table 1, entry 3). On the contrary, in the reaction with Na_2_CO_3_ (Table 1, entry 7), the isomerization did not take place despite the exposure to residual hydroxylamine and sodium carbonate. There are two types of reactions in the oxime synthesis: in situ deprotonation of NH_2_OH·HCl by base and reaction of NH_2_OH and the aldehyde. For the in situ deprotonation, the stoichiometric ratio of NH_2_OH·HCl and NaOH is 1:1, while for the reaction with Na_2_CO_3_, it is 2:1. In all realized experiments with NaOH (except entry 4), the hydroxylamine:hydroxide stoichiometric ratio was higher than 2:1, while for the experiments with Na_2_CO_3_, the hydroxylamine:carbonate ratio was 1.93:1 (almost stoichiometric). We assume that in the second case, complete deprotonation of NH_2_OH·HCl occurred and the resulting reaction mixture was neutral, while in experiments with NaOH, less than half of NH_2_OH·HCl was deprotonated, thus resulting in the acidic conditions. Consequently, residual HCl may cause isomerization. The isomerization of oximes from *anti* (*E*) to *syn* (*Z*) in the presence of strong acids is well known [72,73]. To confirm the hypothesis outlined in this paragraph, additional experiments described later were performed.

In Figure 3, the selected experiments performed using the Pulverisette 6 mill in an agate vessel are compared, namely the one applying LAG procedure (Table 1, entry 5) and the aging one (Table 1, entry 8). When performing LAG in an agate vessel (red, Table 1, entry 5) for 30 minutes, the sample still contained 7% unreacted aldehyde after the reaction, which was almost completely transformed into oximes during the next 20 h. This experiment provided the anticipated result in terms of the amount of *syn* isomer after 4 days, as 84% was measured. However, it seems that LAG enhances the transformation to the *syn* isomer, as 3 h after the reaction, it is the major isomer detected, whereas in the neat aging experiment (blue, Table 1, entry 8), the *anti* isomer was the major isomer after the same amount of time. For the LAG experiment (red, Table 1, entry 5), the aldehyde was not detected at all at the end and the amounts of the oxime isomers were similar to that detected in the final ^1^H-NMR measurement prior to washing.

For the aging experiment (Table 1, entry 8), a significant amount of non-reacted aldehyde was detected after the reaction. It was subsequently consumed during storage, decreasing to less than 10% after one day. However, a complete consumption was not observed. The *anti*-*syn* isomerization took place and the *anti* isomer made up 12% of the product after 4 days. The final content of *anti* isomer after workup was 8%, which is almost the same as in the case of the experiment using LAG procedure (Table 1, entry 5).

The changes in content of aldehyde and *syn* isomer for the aging experiment performed in WC (Table 1, entry 9) with time are presented in the ESI in Figure S6. The reaction did not go to completion and *syn* isomer became greatly major.

After the synthesis and isomerization of oximes in the presence of non-reacted hydroxylamine were investigated in the solid state, these processes were also studied in a solvent by NMR measurements in DMSO. Whereas the presence of solvent facilitates the conversion to oximes (if not finished during milling), the *anti*-*syn* isomerization is more rapid in the solid state. Detailed description of the results can be found in the ESI (Table S3 and the text therein).

In the solution-based syntheses performed in the excess of hydroxylamine (Table S1, entries S1 and S2), *syn* isomer was major. Its content was higher when the mixture was left to isomerize (entry S2). When the same reaction was performed with 5 eq. of both NH_2_OH·HCl and NaOH (Table S1, entry S3), we found that the amount of *syn* isomer after 1 h was only 34%. In this case the reaction mixture was neutral and the *anti* isomer was isolated as the major product. The preference of *anti* isomer was also found after repeating with Hanley’s [55] conditions (59%) and was similarly described by Kawasaki and Somei’s [56] reaction in pyridine (62%). It seems that the main driving force behind the isomerization is the acidity of the reaction mixture. In order to prove this, we subjected pure isomers to both neutral and acidic conditions and studied the isomerization process (see the text below).

After the dissolution of individual isomers and subsequent storage in CDCl_3_ for 7 days (see ESI, Figure S7), the susceptibility to isomerization of pure *anti* oxime into the *syn* one was discovered (equilibrium ratio *syn:anti* 15:85). In contrast, we found that the *syn* isomer is more stable in CDCl_3_ solution and after 7 days *syn*:*anti* equilibrium ratio was 97:3. This is in contradiction with the results of Kawasaki and Somei et al. [56] who observed the transformation of both oxime isomers into the other one and attained an equilibrium significantly favoring the *anti* oxime (*syn*:*anti* 39:61) in both CDCl_3_ and CHCl_3_. The isomerization did not occur in CD_3_OD. They planned to report details of this phenomenon subsequently, but to the best of our knowledge, no report regarding this isomerization was published until now.

**Figure 4:**
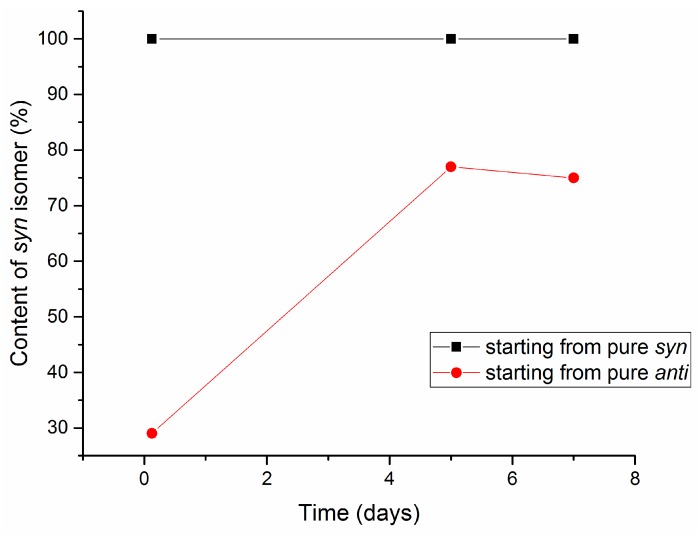
Amounts of *syn* oxime **1a** after milling (Pulverisette 7 Premium Line mill, milling time 20 min) of the pure individual oxime isomers with 10 mol% *p*-TsOH during storage in solid state (the content was determined from ^1^H-NMR spectra presented in the ESI—Figure S8).

In order to investigate the role of acidity in a mechanochemical process, pure isomers were milled with 10 mol% of *p*-TsOH for 20 min (under the same conditions as in the ideal experiment (Table 1, entry 3)). No isomerization was generally observed for *syn* isomer, while 39% of the *anti* isomer was transformed into *syn* one in 1 h after milling and after 7 days, 75% was transformed (Figure 4, corresponding NMR spectra can be found in the ESI in Figure S8). It seems that the *syn* isomer is stable in the solid state under the acidic conditions.

**Figure 5:**
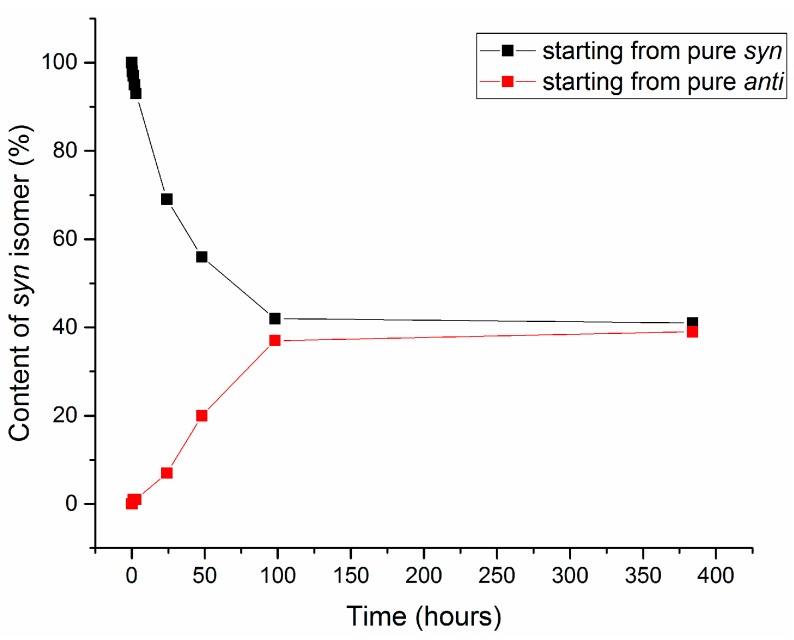
Amounts of *syn* oxime **1a** during storage in DMSO containing 10 mol% *p*-TsOH starting from pure isomers (the content was determined from ^1^H-NMR spectra presented in the ESI—Figure S9).

To compare the behavior of individual isomers with the solid-state isomerization experiments under the acidic conditions, the behavior of separated isomers in DMSO-*d*_6_ with 10 mol% *p*-TsOH was investigated. In this case, each isomer changed to the other one and an equilibrium with ratio of *syn*:*anti* ratio 40:60 was slowly achieved (Figure 5, corresponding NMR spectra can be found in the ESI in Figure S9), being very close to those reported in [56]. From these results two major points regarding isomerization under acidic conditions can be claimed: (i) on the contrary to the mechanochemical approach, the isomerization of the *syn* isomer takes place in solution and (ii) whereas the formation of *anti* isomer is favored in solution, *syn* isomer is preferred in the solid state. This brings us to the specificity of mechanochemistry, which is known to provide different results than if the same reaction is performed in a traditional way [24]. Further effort will be necessary to enlighten our observations.

The detailed characterization of the pure 1-methoxyindole-3-carboxaldehyde oximes using various methods can be found in the Electronic Supplementary Information (ESI), namely the complete set of NMR spectra of both isomers (Figures S10–S19), proofs of configuration (*syn* and *anti*, Figure S20 and Table S4–S6), X-ray diffraction patterns (Figure S21), FT-infrared spectra (Figure S22), melting temperature analysis, optical microscopy images (Figure S23) and transmission electron microscopy images (Figure S24).

### 2.4. Substrate Scope in the Oximation-Isomerization Reactions

In order to check if the proposed approach could be more universal, we have tested it with different substituents on nitrogen. Namely non-substituted (**2**), 1-methyl- (**3**) and 1-acetyl-indole-3-carboxaldehyde (**4**) were used for the reactions using either NaOH or Na_2_CO_3_ as a base.

The results both in terms of reaction completion and isomerization are summarized in Table 2. The isolated yields are reported after extraction with water (ethyl acetate was not applied in this case). For this purpose, the reactions with 1-methoxyindole-3-carboxaldehyde were also repeated. The reaction conditions and yields for the solution-based approaches reported in literature are also provided for comparison.

In all cases the content of aldehyde is almost negligible, which means that the proposed methodology can be used in a more universal fashion for the transformation of different *N*-substituted indole-based aldehydes into corresponding oximes. The content of individual isomers was significantly different both in terms of a base and substituent used.

For the reaction of 1-methoxy analog with NaOH, the washing with water leads to the significant prevalence of the *syn* isomer already after the reaction. In comparison with the relevant studies, our approach does not require high [57] or low temperature [55], or pyridine solvent [56].

The reactions of non-substituted aldehyde yielded only the *syn* isomer, regardless of the base used, which is in accordance with the available literature reporting the formation of only one isomer, although its configuration is not assigned therein [49,51,52]. The reaction times in the most relevant papers are comparable with our results, however, in most cases elevated temperature was necessary for the reaction completion within a reasonable time [49,51].

1-methylindole-3-carboxaldehyde oxime has shown the similar trend as its methoxy-substituted analog in terms of *anti*-*syn* isomerization when performing the reaction with NaOH. After the reaction, the *syn* isomer was already major (53%) and further storage of reaction mixture resulted in an increase of its content to 74% after more than 10 days. Its prevalence is lower than for the methoxy analog. The *anti*:*syn* ratio for the reaction performed with Na_2_CO_3_ did not change during storage, similarly to the methoxy analog. The content of the major *anti* isomer was 67%, which was higher than in the case of methoxy-substituted product (57%).

In the case of the electron-withdrawing acetyl group, under the given experimental conditions, in addition to the formation of the **4a** *syn* and *anti* isomers, the product deacetylated to form the *syn*-**2a**. The extent of deacetylation was greater when the reaction was performed with Na_2_CO_3_, where the contents of deacetylated oxime **2a** were 48% and 100% in the sample washed immediately after reaction and after isomerization, respectively. The acidic conditions (in this experiment, the excess of NH_2_OH·HCl over NaOH was used, similarly to entry 3 in Table 1) did not result in as much deacetylation, although a considerable amount was evident after isomerization (21%). The *anti*-*syn* isomerization was observed when the reaction was performed in NaOH, however, after 10 days, the *anti* isomer was still the major isomer, so the process is much slower in comparison with the experiments where electron-donating groups were present. The reaction with Na_2_CO_3_ resulted in the formation of almost pure *anti* isomer (50%) and a significant amount of deacetylated *syn*-**2a** (48%). The product was completely transformed into deacetylated analog *syn*-**2a** after 10 days. It seems that the isomerization process is accompanied by deacetylation in this case. The relevant literature reports used sodium acetate as a base and either higher temperature [54] or longer time [53] was necessary for the reaction completion.

From the available NMR data (chemical shifts of carbonyl carbon) it is clear that whereas the presence of acetyl group increases the electron deficiency on the carbonyl carbon [74], methyl and methoxy groups compensate it by donating electrons [75,76]. Based on this, the following order can be worked out (starting from the largest electron-donating effect on carbonyl carbon): methoxy > methyl > H > acetyl. In the case of electron-donating substituents, the *anti*-*syn* isomerization occurs, being more significant for the 1-methoxy-substituted oxime **1a** than for the 1-methyl-substituted analog (**3a**), thus following the proposed order. However, the non-substituted oxime **2a** was present only in the *syn* configuration already after the reaction, and the acetyl-substituted oxime **4a** exhibited deacetylation and slower *anti*-*syn* isomerization when compared to the analogs containing electron-donating groups. Thus the results obtained for **2a** and **4a** do not follow the proposed order. Further research is necessary to clarify the observed reactivity and isomerization but the substituent evidently plays a decisive role here.

The isolated yields are higher for the reactions performed with NaOH, being almost quantitative. In the case of reactions performed with Na_2_CO_3_, the mixture seems to stick to the chamber walls and it is not possible to extract it quantitatively just by washing with water.

## 3. Materials and Methods

### 3.1. Chemicals

The following chemicals were used for the experiments: indoline (99%, Alfa Aesar, Haverhill, MA, USA), Na_2_WO_4_·2H_2_O (98.5%, Centralchem, Banská Bystrica, Slovakia), K_2_CO_3_ (98%, Centralchem, Slovakia), POCl_3_ (99%, Merck, Darmstadt, Germany) NH_2_OH·HCl (99%, Centralchem, Slovakia), NaOH (98%, Centralchem, Slovakia) and Na_2_CO_3_ (98%, Centralchem, Slovakia), indole-3-carboxaldehyde (97%, Merck, Germany).

### 3.2. Preparation of Aldehydes

**Scheme 2:**
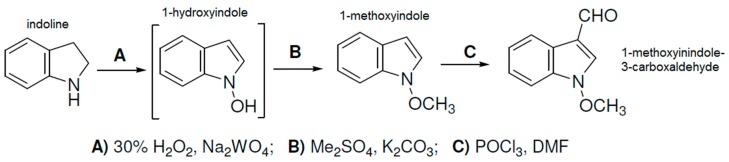
Somei’s methodology for the synthesis of 1-methoxyindol-3-carboxyaldehyde [42,43,44].

1-Methoxyindole-3-carboxaldehyde was prepared by a classical approach from indoline using a Somei’s methodology [42,43,44] (Scheme 2):

1-Methylindole-3-carboxaldehyde was prepared according to the procedure reported in [77]. 1-Acetylindole-3-carboxaldehyde was prepared according to the procedure reported in [53].

### 3.3. Experimental Procedure for Mechanochemical Synthesis

In the experiments performed with tungsten carbide milling media, a planetary ball mill Pulverisette 7 premium line (Fritsch, Idar-Oberstein, Germany) with a special 80 mL milling vessel equipped with a sensor for measuring gas pressure and temperature (EasyGTM, Fritsch, Germany) was used. Eighteen WC milling balls with diameter 10 mm were used and the whole mass of reactants was 1.9699 g. In the case of milling in agate, a planetary ball mill Pulverisette 6 (Fritsch, Germany) and an agate vessel of 250 mL volume were applied. In this case, 39 agate milling balls with diameter 10 mm were used and the whole mass of reactants was 1.3613 g. The number of balls and mass of reactants in both cases was selected in order to maintain ball-to-powder ratio 71. The atmosphere in both cases was air. All experiments were performed at a milling speed of 500 rpm. Further details regarding the experimental conditions are listed in Table 1. After milling, the reaction mixtures were either scraped from the milling chamber manually, or extracted with ethyl acetate (30 mL). The latter was done for the entries 3, 4, and 6.

The mixtures were then washed with distilled water, in order to remove the residual hydroxylamine hydrochloride. For 1.4 g of reaction mixture, 70 mL of distilled water was used. This ratio was maintained also when a different mass was used. The washed mixtures were then filtered, dried at 75 °C, and stored.

When the progress of the isomerization process was targeted, the solid mixtures were left standing in a closed glass vessel (not evacuated) for 7–8 days prior to washing.

For the experiments applying other aldehydes (non-substituted, 1-methyl and 1-acetyl), the experimental conditions from entries 3 and 7 from Table 1 were applied. In some cases, some powder was stuck to the lid of the milling chamber. This was excluded from further analysis. The powder after the reaction was separated into two fractions: 0.5 g was extracted and stored for the study of possible isomerization for more than 10 days and the rest of powder was immediately extracted from the chamber using distilled water and washed under the conditions described earlier. The same was done with the isomerizing powder after more than 10 days. In order to investigate the effect of immediate water washing, this approach was used also for the 1-methoxy analog.

For the thin layer chromatography (TLC) analysis, the Silica gel 60 F_254_ aluminum sheets (Merck, Germany) were used. A diethylether:hexane 4:1 mixture was used as the eluent. The relative content of the isomers was evaluated densitometrically by comparing the intensities of the corresponding spots using the software NIH ImageJ 1.48.

Column chromatography with Silica gel 60 (0.040–0.063 mm, Merck, Germany) on a glass column with the diameter 20 mm and internal volume 400 mL was used for the separation of the isomers.

For all experiments, the product, after washing with water, was purified from media wear using flash chromatography over silica gel. The product absorbed on the silica gel was eluted using hexane:ethyl acetate 1:1.

With the aim to separate and characterize both isomers of 1-methoxyindole-3-carboxaldehyde oxime, the product of the milling process (Table 1, entry 4) was extracted with ethyl acetate, then washed with water (25 mL) and brine (10 mL), dried over Na_2_SO_4_ and filtered. The washed product obtained directly from the reaction vessel was dissolved in this filtrate and the solvent evaporated with small amount of silica gel. The residue after evaporation was subjected to chromatographic separation over silica gel (hexane/ethyl acetate 2:1). The individual isomers were then crystallized (dichloromethane:hexane).

### 3.4. Experimental Procedure for Solution-Based Synthesis

To a solution of 1-methoxyindol-3-carboxaldehyde (0.25 mmol) in ethanol (0.6 mL) NH_2_OH·HCl (1.25 mmol, 5 eq.) and NaOH (0.5 mmol, 2 eq. for Table S1, Entry S1 and S2 and 1.25 mmol, 5 eq. for Table S1, Entry S3) in water (0.32 mL) at 0 °C were added. The reaction mixture was stirred at room temperature for 1 h (in the case of Entry S2, it was left without further processing at room temperature for 7 days), then diluted with ethyl acetate (10 mL), washed with water and brine, dried and evaporated.

### 3.5. Isolated Yield Calculation

The isolated yield was calculated as follows:

(1) $$\mathrm{Isolated}\mathrm{yield}\left(\%\right)=\left(\frac{m_{i}}{m_{T}}\right)\cdot 100$$

where m_i_ is the isolated mass of the product after extraction and subsequent purification by flash chromatography and m_T_ is the theoretical yield.

### 3.6. Spectral Data for the Products

#### 3.6.1. *Anti*-1-methoxyindole-3-carboxaldehyde Oxime (*anti*-**1a**)

Yield: 0.2073 g, 31% (ref. [56] 39%). Melting point: 93–95 °C (dicholomethane/ hexane) (ref. [56] 146–147 °C). White crystals. XRD: *2θ* 11.3, 12.5, 13.8, 17.8, 20.8, 22.6, 27.8, 29.3, 34.9, 36.1, 42.2, 49.6, 57.3°. IR: *ν* 3242, 3186, 3121, 2986, 2939, 2897, 1640, 1541, 1447, 1377, 1346, 1327, 1290, 1244, 1163, 1109, 1043 941, 806, 716 cm^−1^. ^1^H-NMR (600 MHz, CDCl_3_): δ = 8.29 (s, 1H, H-8), 8.06 (d, 1H, *J* 8.1 Hz, H-4), 7.48 (s, 1H, H-2), 7.45 (d, 1H, *J* 8.3 Hz, H-7), 7.32 (ddd, 1H, *J* 8.2, 7.0, 1.1 Hz, H-6), 7.23 (ddd, 1H, *J* 8.0, 7.1, 1.0 Hz, H-5), 4.12 (s, 3H, CH_3_O). ^13^C-NMR (151 MHz, CDCl_3_): 145.4 (C-8), 132.6 (C-7a), 124.8 (C-2), 123.7 (C-6), 122.2 (C-4), 121.6 (C-5), 121.3 (C-3a), 108.4 (C-7), 105.6 (C-3), 66.3 (CH_3_O). ^1^H-NMR (600 MHz, DMSO): δ = 10.69 (s, 1H, H-10), 8.21 (s, 1H, H-8), 8.00 (d, 1H, *J* 8.1 Hz, H-4), 7.95 (s, 1H, H-2), 7.49 (d, 1H, *J* 8.2 Hz, H-7), 7.29 (ddd, 1H, *J* 8.2, 7.0, 1.2 Hz, H-6), 7.17 (ddd, 1H, *J* 8.0, 7.1, 1.0 Hz, H-5), 4.09 (s, 3H, CH_3_O). ^13^C-NMR (151 MHz, DMSO): 143.6 (C-8), 132.2 (C-7a), 125.9 (C-2), 123.3 (C-6), 122.0 (C-4), 121.0 (C-5), 120.6 (C-3a), 108.5 (C-7), 105.9 (C-3), 66.2 (CH_3_O) ppm. ^1^J_C8H8_ = 161.4 Hz. *R_f_* value (diethylether:hexane 4:1): 0.69.

#### 3.6.2. *Syn*-1-methoxyindole-3-carboxyaldehyde Oxime (*syn*-**1a**)

Yield: 0.3519 g, 56% (ref. [56] 59%). Melting point: 137–139 °C (dicholomethane/ hexane) (ref. [56] 98.5–99.5 °C). White crystals. XRD: *2θ* 11.9, 15.7, 16.5, 17.7, 18.6, 22.4, 24.0, 24.5, 30.5°. IR: *ν* 3151, 3063, 3018, 2935, 2824, 1639, 1512, 1327, 1229, 1177, 1096, 1024, 941, 746 cm^−1^. ^1^H-NMR (600 MHz, CDCl_3_): δ = 8.40 (s, 1H, H-2), 7.79 (s, 1H, H-8), 7.79 (d, 1H, *J* 7.9 Hz, H-4), 7.49 (d, 1H, *J* 8.2 Hz, H-7), 7.33 (t, 1H, *J* 7.5 Hz, H-6), 7.25 (t, 1H, *J* 7.3 Hz, H-5), 4.16 (s, 3H, CH_3_O). ^13^C-NMR (151 MHz, CDCl_3_): 139.5 (C-8), 130.7 (C-7a), 128.5 (C-2), 123.2 (C-6), 123.2 (C-3a), 121.4 (C-5), 118.4 (C-4), 108.5 (C-7), 101.7 (C-3), 66.4 (CH_3_O). ^1^H-NMR (600 MHz, DMSO): δ = 11.42 (s, 1H, H-10), 8.42 (s, 1H, H-2), 7.94 (d, 1H, *J* 8.0 Hz, H-4), 7.83 (s, 1H, H-8), 7.52 (d, 1H, *J* 8.2 Hz, H-7), 7.30 (ddd, 1H, *J 8.2,* 7.1, 1.1 Hz, H-6), 7.19 (ddd, 1H, *J* 8.0, 7.0, 1.0 Hz, H-5), 4.13 (s, 3H, CH_3_O). ^13^C-NMR (151 MHz, DMSO): 137.5 (C-8), 130.1 (C-7a), 127.6 (C-2), 123.0 (C-6), 122.5 (C-3a), 120.8 (C-5), 118.9 (C-4), 108.4 (C-7), 102.2 (C-3), 66.4 (CH_3_O) ppm. ^1^J_C8H8_ = 172.2 Hz. *R_f_* value (diethylether:hexane 4:1): 0.53.

#### 3.6.3. *Syn*-indole-3-carboxyaldehyde Oxime (*syn*-**2a**)

Yield: 0.4972 g, 85%. Melting point: 179–181 °C (acetone/hexane) (ref. [50] 182 °C, chloroform). White crystals. ^1^H-NMR (400 MHz, DMSO-*d*_6_): *δ* = 11.58 (s, 1H, H-1), 11.19 (s, 1H, H-10), 8.23 (d, 1H, *J* = 2.7 Hz, H-2), 7.86 (d, 1H, *J* = 7.8 Hz, H-4), 7.79 (s, 1H, H-8), 7.44 (d, 1H, *J* = 8.0 Hz, H-7), 7.17 (ddd, 1H, *J* = 8.0, 7.0, 1.3 Hz, H-6), 7.10 (ddd, 1H, *J* = 8.0, 7.0, 1.2 Hz, H-5) ppm. ^13^C-NMR (100 MHz, DMSO-*d*_6_): *δ* = 138.4 (C-8), 134.9 (C-7a), 130.5 (C-2), 126.2 (C-3a), 121.9 (C-6), 119.9 (C-5), 118.2 (C-4), 111.8 (C-7), 106.3 (C-3) ppm. ^1^J_C8H8_ = 169 Hz. *R_f_* value (hexane:acetone 2:1): 0.19.

#### 3.6.4. *Syn* and *anti*-1-methylindole-3-carboxyaldehyde Oxime (**3a**)

Melting point: 134–136 °C (dichloromethane/hexane). White crystals.

Spectral data for *anti*-**3a**: ^1^H-NMR (400 MHz, DMSO-*d*_6_): *δ* = 10.50 (s, 1H, H-10), 8.21 (s, 1H, H-8), 7.96 (d, 1H, *J* = 7.9 Hz, H-4), 7.57 (s, 1H, H-2), 7.43 (d, 1H, *J* = 8.2 Hz, H-7), 7.21 (ddd, 1H, *J* = 8.2, 7.0, 1.2 Hz, H-6), 7.13 (m, 1H, H-5) ppm. ^13^C-NMR (100 MHz, DMSO-*d*_6_): *δ* = 144.0 (C-8), 137.2 (C-7a), 131.9 (C-2), 124.5 (C-3a), 121.8 (C-6), 121.4 (C-4), 120.1 (C-5), 109.9 (C-7), 108.5 (C-3), 32.5 (Me) ppm. *R_f_* value (hexane:ethylacetate 2:1): 0.42.

Spectral data for *syn-***3a**: ^1^H-NMR (400 MHz, DMSO-*d*_6_): *δ* = 11.22 (s, 1H, H-10), 8.22 (s, 1H, H-2), 7.83 (d, 1H, *J* = 7.8 Hz, H-4), 7.73 (s, 1H, H-8), 7.45 (d, 1H, *J* = 8.2 Hz, H-7), 7.21 (ddd, 1H, *J* = 8.2, 7.0, 1.2 Hz, H-6), 7.13 (m, 1H, H-5) ppm. ^13^C-NMR (100 MHz, DMSO-*d*_6_): *δ* = 138.5 (C-8), 135.3 (C-7a), 134.2 (C-2), 126.5 (C-3a), 122.2 (C-6), 119.9 (C-5), 118.2 (C-4), 109.9 (C-7), 105.6 (C-3), 32.6 (Me) ppm. *R_f_* value (hexane: ethylacetate 2:1): 0.31.

#### 3.6.5. *Anti* and *syn*-1-acetylindole-3-carboxyaldehyde Oxime (**4a**)

White powder (in mixture with *syn*-**2a**).

Spectral data for *anti*-**4a**: ^1^H-NMR (400 MHz, DMSO-*d*_6_): *δ* = 11.98 (s, 1H, H-10), 8.58 (s, 1H, H-2), 8.36 (d, 1H, *J* = 8.2 Hz, H-7), 7.97 (d, 1H, *J* = 7.8 Hz, H-4), 7.92 (s, 1H, H-8), 7.37 (m, 2H, H-5, H-6) ppm. ^13^C-NMR (100 MHz, DMSO-*d*_6_): *δ* = 169.5 (C-11), 136.9 (C-8), 134.0 (C-7a), 130.4 (C-2), 128.5 (C-3a), 125.3 (C-6), 123.8 (C-5), 119.0 (C-4), 115.9 (C-7), 110.9 (C-3), 23.9 (Me) ppm. ^1^J_C8H8_ = 163.2 Hz. *R_f_* value (hexane: ethylacetate 2:1): 0.33.

Spectral data for *syn*-**4a**: ^1^H-NMR (400 MHz, DMSO-*d*_6_): *δ* = 11.15 (s, 1H, H-10), 8.36 (d, 1H, *J* = 8.2 Hz, H-7), 8.29 (s, 1H, H-8), 8.16 (s, 1H, H-2), 8.08 (d, 1H, *J* = 7.4 Hz, H-4), 7.37 (m, 2H, H-5, H-6) ppm. ^13^C-NMR (100 MHz, DMSO-*d*_6_): *δ* = 169.9 (C-11), 143.3 (C-8), 135.7 (C-7a), 129.1 (C-2), 126.8 (C-3a), 125.6 (C-6), 124.0 (C-5), 122.1 (C-4), 115.9 (C-7), 115.3 (C-3), 23.8 (Me) ppm. ^1^J_C8H8_ = 174.4 Hz. *R_f_* value (hexane: ethylacetate 2:1): 0.28.

## 4. Conclusions

The successful mechanochemical conversion of four *N*-substituted indole-3-carboxaldehydes into the corresponding oximes was achieved in a safe solvent-free mode, although the 1-acetyl-analog was partly deacetylated during the reaction. This approach offers safe handling of dangerous hydroxylamine hydrochloride. Under optimal conditions, the product containing nearly pure *syn* isomer can be synthesized for the 1-methoxy analog after the solvent-free milling of the reagents for 20 min in a tungsten carbide milling vessel at 500 rpm. When the residual hydroxylamine hydrochloride is not immediately washed out after the mechanochemical process (the acidic conditions are maintained), the *anti* oxime is nearly completely transformed into the *syn* isomer within three days. The same transformation was also observed for 1-methyl analog. However, if the conditions are neutral (balanced stoichiometry between hydroxylamine and base), this transformation is not observed. The main force behind the isomerization is the acidity of the reaction mixture, however, mechanochemistry enhances this process. Under acidic conditions, the isomerization favors the opposite isomers for the milled mixture and in solution. Whereas in the first case *syn* isomer is preferred, in solution an equilibrium favoring the *anti* one is reached. The study of such isomerization is novel and represents another stimulus for the organic mechanochemists. This study shows that mechanochemical syntheses can be applied for the realization of an environmentally friendly syntheses of diastereoisomerically pure compounds, which will facilitate the production of synthons for the development of new pharmaceuticals. The environmental and personal risk is much lower in comparison with the solvent-based synthesis methods.

## Figures and Tables

**Table 1:** Basic experimental conditions used in the synthesis of 1-methoxyindole-3-carboxaldehyde oxime and the reactions outcome. The different reaction conditions are marked by color: green color: ball milling in Pulverisette 7 premium line mill, WC vessel (80 mL) and balls (in most cases 18 pieces, ⌀ 10 mm, weight of 1 ball 7.7 g); blue color: ball milling in Pulverisette 6 mill, agate vessel (250 mL) and balls (50 pieces, ⌀ 10 mm, weight of 1 ball 2.4 g). The ideal experiment is marked in bold. The milling time is reported for the final mixtures from which the NMR spectra were recorded. Until this time, the reaction progress was monitored by TLC chromatography. The inconsistencies between the conversion and isolated yield values come only from the ineffectiveness of manual extraction of reaction mixture from the milling vessel.

Entry	Equivalents		Milling/Stirring Time (min)	Conversion from NMR	*Syn*:*anti* Oxime Ratio	Isolated Yield
	NH_2_OH·HCl	NaOH				
1	3.1	2.5	30	100	35:65	78
2	5	2.5	20	100	98:2	72
3 *^a^*	5	2	20	100	97:3	94
4 *^a^*	5	5	20	100	43:57	93
5 *^b^*	5	2	30	100	87:13	20
6 *^ac^*	5	2	40	100	91:9	80
7 *^d^*	5.2	2.7	40	100	30:70	45
**Aging experiments**						
8	5	2	5	97 *^e^*	95:5	25
9	5	2	0.25	85 *^e^*	89:11	65

*^a^* The powder from the vessel was thoroughly extracted with ethyl acetate, in order to isolate as much product as possible. *^b^* Liquid assisted grinding (LAG) through the addition of a few drops of methanol was performed, η value = 0.022 µL/mg. *^c^* The reaction mixture was washed with distilled water and the products were isolated immediately after its termination. *^d^* Instead of NaOH, Na_2_CO_3_ was used as a base. *^e^* The indicated conversion was not measured immediately after the short milling time indicated in corresponding column, but 10 days after reaction and after washing procedure, so there was plenty of time for solid-state reaction to progress also outside milling device (for more details, see Figure 2 and Figure 3). It was found that the reaction proceeds to some extent also in DMSO solution (see Table S2). The great prevalence of *anti* isomer (on the contrary to the mechanochemical experiments) can be seen. In order to minimize the contribution from solution reaction, the measurements were done quickly (ca. 2 min after dissolution).

**Table 2:** Conversions, isolated yields and *syn*:*anti* oxime ratios immediately after reaction and after isomerization. The basic experimental conditions, together with yields reported in relevant literature are provided for comparison. In a reference column, either a link to corresponding figures in the supplementary information (NMR spectra, Figures S25–S35), or a literature reference is provided. For all experiments, the conditions for entries 3 or 7 in Table 1 (when the reaction was performed with NaOH or Na_2_CO_3_, respectively), were used.

N-R		Conversion from NMR	*Syn*:*anti* Ratio		Isolated Yield	Reference
	Base		After Reaction	After Isomerization		
**OCH_3_**	NaOH	99	81:19	97:3	78	Figure S26
	Na_2_CO_3_	99	43:57	43:57	67	
	Na_2_CO_3_				50	[55]
	pyridine		60:40 *^a^*		98	[56]
	Na_2_CO_3_				99	[57]
**H**	NaOH	100	100:0	100:0	95	Figure S27–S29
	Na_2_CO_3_	100	100:0	100:0	76	
	NaOH				91	[49]
	NaOH				62	[50]
	Na_2_CO_3_				78	[51]
	Na_2_CO_3_				98	[52]
**CH_3_**	NaOH	98	53:47	74:26	85	Figure S30–S32
	Na_2_CO_3_	100	32:68	33:67	71	
	NaOH		68:32 *^b^*		86	[50]
	Na_2_CO_3_				100	[52]
**CO-CH_3_**	NaOH	100	19:70:12 *^c^*	34:45:21 *^c^*	96	Figure S33–S35
	Na_2_CO_3_	100	2:50:48 *^c^*	0:0:100 *^c^*	71	
	NaOAc				88	[53]
	NaOAc				84	[54]

^a^ The ratios were re-calculated from the reported yields. ^b^ The configuration of isomers is not assigned in the paper, just the ratio is reported. ^c^ The ratios in this case are reported as follows: *syn*-**4a**:*anti*-**4a**:*syn*-**2a** (deacetylated analog).

## Supplementary Materials

The following are available online. Supporting information for this paper contain: NMR spectra reporting on the conversion of reaction and isomerization (both in neutral and acidic conditions); information about pressure and temperature of gas in the milling chamber during the process; photographs of the TLC plates of the isomerization mixture; changes in ratios of 1-methoxyindole-3-carboxaldehyde oxime isomers calculated from the intensity of TLC spots with time for selected experiment; and characterization results of pure 1-methoxyindole-3-carboxaldehyde oxime isomers, namely ^1^H, ^13^C and ^15^N NMR spectra, XRD patterns, FTIR spectra, melting temperatures description, optical microscopy analysis, and transmission electron microscopy analysis. Also few appropriate references are provided [23,55,56,60,78,79,80].
