# Supplementary material for: Mechanochemical Synthesis and Isomerization of N-Substituted Indole-3-carboxaldehyde Oximes
Source: Molecules. 2019 Sep 14;24(18):3347. doi: 10.3390/molecules24183347 (PMC6766794; doi:10.3390/molecules24183347)
Supplement: Supplementary file 1 [file molecules-24-03347-s001.pdf]

Electronic Supplementary Information

for the paper

Mechanochemical synthesis and isomerization of 1-methoxyindole-3-carboxaldehyde oxime

by

Matej Baláž, Zuzana Kudličková, Mária Vilková, Ján Imrich, Ľudmila Balážová, Nina Daneu

1. Analysis of the reaction progress

Initially, the procedure reported in [1] using only mortar and pestle was tried. The reaction did not go to completion as it barely progressed under these conditions (Table S1, ref. [1]). This may be due to the fact that the starting aldehyde contains an indole ring in its structure and more energy is required for the reaction to proceed. The electron-donating resonance effect of indole nitrogen decreases the electrophilicity of carbonyl carbon [2]. In Table S1, the results for the solution-based experiments with NaOH (entries S1, S2 and S3) and Na_2CO_3 (replicating the procedure reported in ref. [3] are provided.

Table S1: Basic experimental conditions used in the solution-based syntheses of 1-methoxyindole-3-carboxaldehyde oxime and the reactions outcome.

Entry	Equivalents		Milling/stirring time (min)	Conversion from NMR	*Syn* : *anti* oxime ratio	Isolated yield
	NH_2OH .HCl	NaOH				
ref. [1],[a]	1.2	1.2	8	41	20 : 80	55
S1[b]	5	2	60	100	59 : 41	95
S2	5	2	60	100	77 : 33	93
S3[b]	5	5	60	100	34 : 66	93
ref. [3],[ac]	5.2	2.7	120	99	40 : 60	98

[a]Conversion and isolated yield was determined from crude product after evaporation ethyl acetate extract.

[b]The reaction mixture was washed with distilled water and the products were isolated immediately after its termination.

[c]Instead of NaOH, Na_2CO_3 was used as a base.

Table S2: Amounts of aldehyde and both oxime isomers upon dissolution of the starting reaction mixture in DMSO after given amount of time.

Time (min)	aldehyde	*syn* oxime	*anti* oxime
5	22	15	63
30	15	17	68
60	14	17	69
120	12	18	70

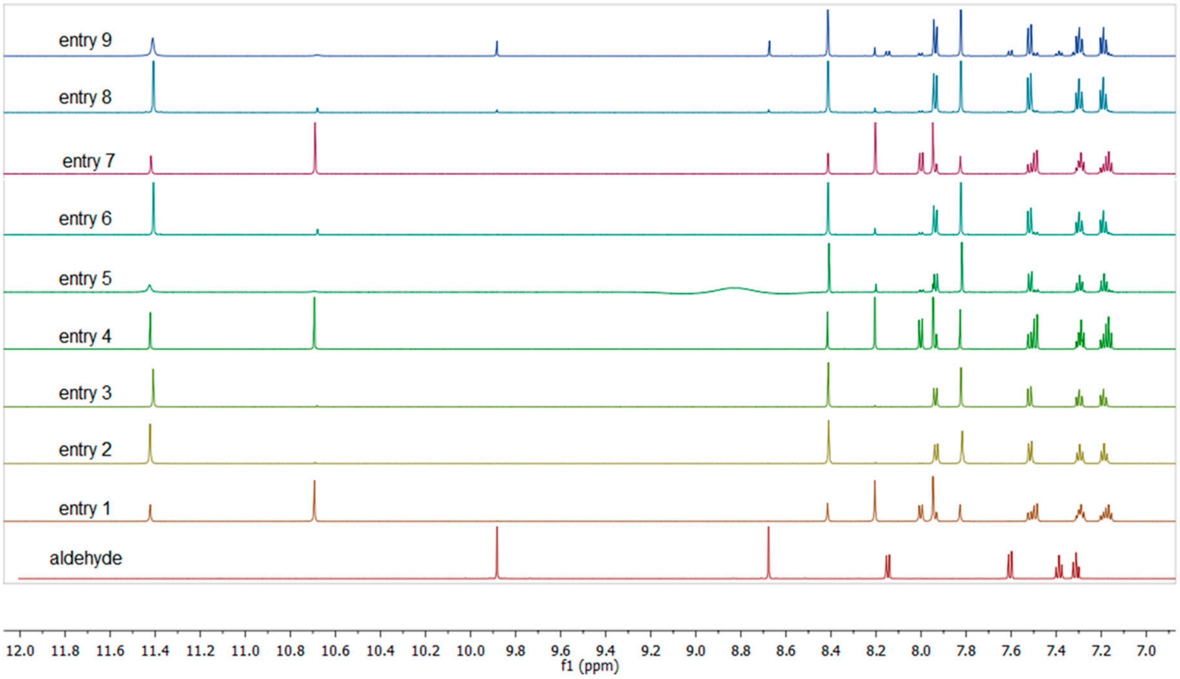

Fig. S1: ^{1}H NMR spectra of all reaction mixtures after removal of residual $NH_2OH.HCl$ and subsequent purification. The experimental conditions can be found in Table 1 in the main body of the paper.

2. Gas and pressure changes during milling

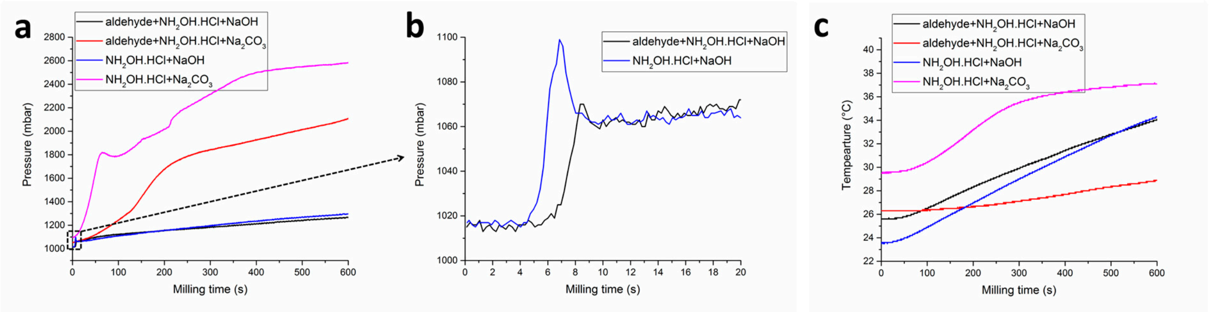

Fig. S2: Gas pressure and temperature changes during milling: (a) pressure changes during the first 10 minutes; (b) pressure changes during the first 20 seconds for the experiments with NaOH; (c) temperature changes during the first 10 minutes. Used experimental conditions: P7 mill, WC milling material. For the experiments involving aldehyde, 5 eq. $NH_2OH.HCl$ and 2 eq. NaOH or 5.2 eq. $NH_2OH.HCl$ and 2.7 eq Na_2CO_3 were used. For the experiments without aldehyde, the masses of base and hydroxylamine were recalculated in order to maintain the ball-to-powder ratio.

From the Fig. S2a, in which the pressure changes are presented, it can be seen that there is a significant difference between the experiments performed with NaOH and Na_2CO_3. At first, it was hypothesized that maybe the reaction including aldehyde proceeds through an exotherm, however, as more significant changes were evidenced without aldehyde, it is clear that these events happen only as a result of the reaction between the base and $NH_2OH.HCl$. In general, there are two types of reactions in the described system: (i) *in situ* deprotonation of $NH_2OH.HCl$ by NaOH or Na_2CO_3 and (ii) reaction of NH_2OH and the aldehyde. Deprotonation is typically an exothermic step and in this case results in the formation of NH_2OH, which is a volatile reagent. Also water is released, which additionally contributes to the pressure build-up. The deprotonation process seems to be much faster in the case of the reaction with NaOH. Therefore, the first 20 seconds of milling are shown in more detail in Fig. S2b. A dramatic increase in the pressure after 10 seconds is evidenced for the mixture containing aldehyde. The process started even earlier without aldehyde and the overall pressure was even higher, however, its value dropped to the one of the reaction including aldehyde after the maximum. After this stage, only a gradual increase of pressure was observed throughout the whole milling process, which is a consequence of milling. No further events could be detected.

In the case of deprotonation using Na_2CO_3, apart from the above products, gaseous CO_2 should be abruptly released, which most probably leads to much higher pressure in the jar. This is confirmed, as the dramatic pressure increase took much longer and the pressure reached significantly higher values. The pressure values were also significantly higher for the mixture without aldehyde, but this time the difference in pressure values persisted also after the dramatic increase.

The temperature curves (Fig. S2c) do not show any interesting events, although it has to be noted that the temperature increases more rapidly in the experiments involving NaOH.

3. Aging experiments

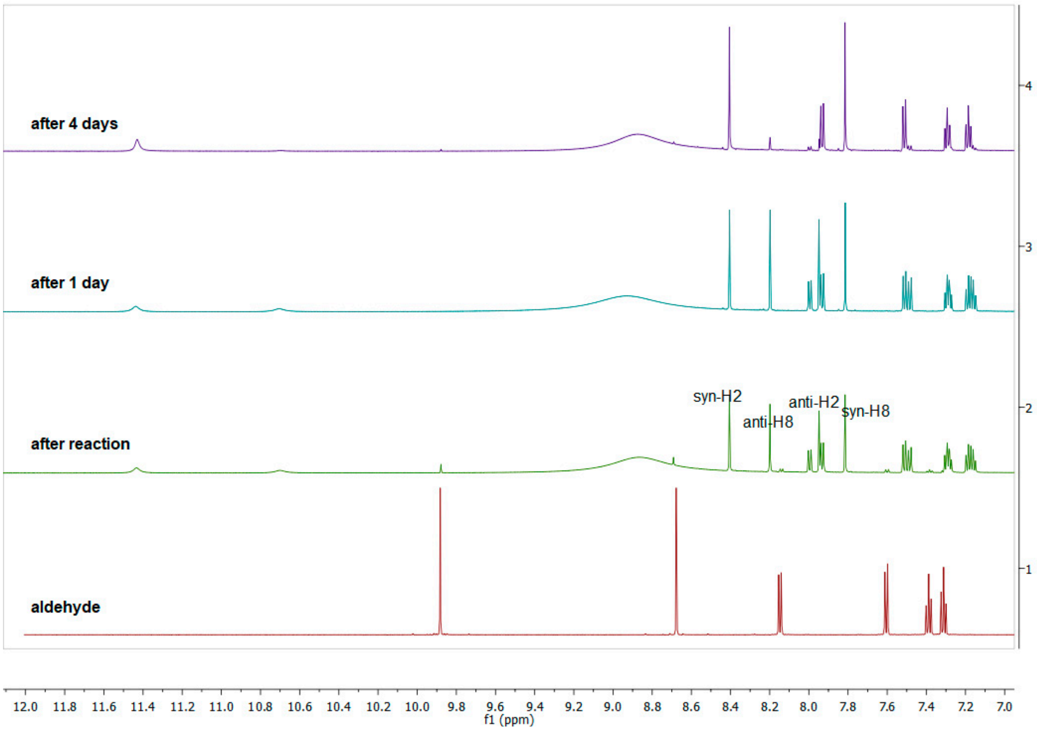

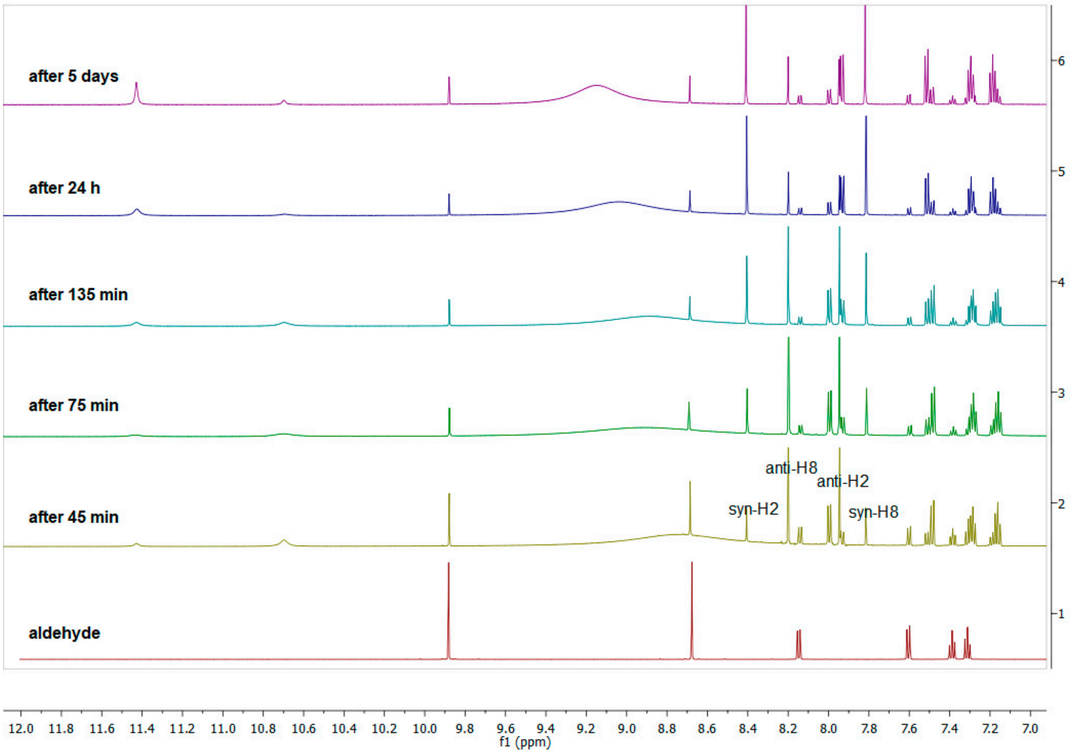

Fig. S3: Reaction progress during the diffusion reaction as monitored by the ^{1}H NMR spectroscopy. Top (Table 1, entry 8): ball-milled in Pulverisette 6, milling material agate, time 5 min. Bottom (Table 1, entry 9): ball-milled in Pulverisette 7, milling material WC, time 15 s. In both cases 1-methoxyindole-3-carboxaldehyde was ball-milled with 5 eq. $NH_2OH.HCl$ and 2 eq. NaOH.

4. Analysis of isomerization

4.1 Post-milling isomerization

The isomerization was pursued in detail for the experiment performed under similar conditions as the entry 6 in Table 1 (except the ethylacetate wash and the fact that the reaction mixture was left to isomerize). The photographs of TLC plates after different times can be found in the ESI (Fig. S4).

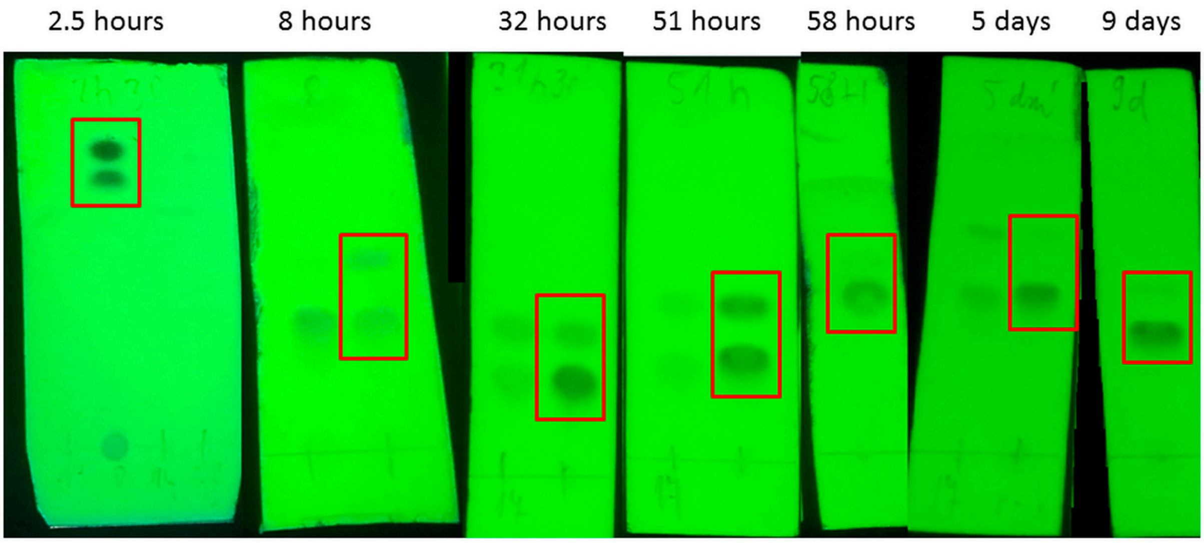

Fig. S4: Isomerization of *anti*-1-methoxyindole-3-carboxaldehyde oxime into *syn* isomer after the experiment labeled as entry 6 in Table 1 (5 eq $NH_2OH.HCl$, 2 eq NaOH, milling speed 500 rpm, BPR 71, reaction time 40 min, agate vessel and balls, Pulverisette 6 mill) pursued by comparing TLC plates (diethylether/hexane 4:1). Time of transformation is provided in figure and the stains corresponding to oximes are marked: *syn*- bottom (Rf = 0.29); *anti*- top (Rf = 0.44).

Using NMR (after separation of isomers), it was later determined that the top stain corresponds to the *anti* isomer and the bottom to *syn* one. It can be clearly seen that the top spot corresponding to the *anti* isomer disappeared with time. It was almost undetectable at 58 h after reaction, and after 5 days, equilibrium was established. The relative intensity of the spots on the TLC plates attributed to the oxime isomers was evaluated using ImageJ software. The results are presented in Fig. S5.

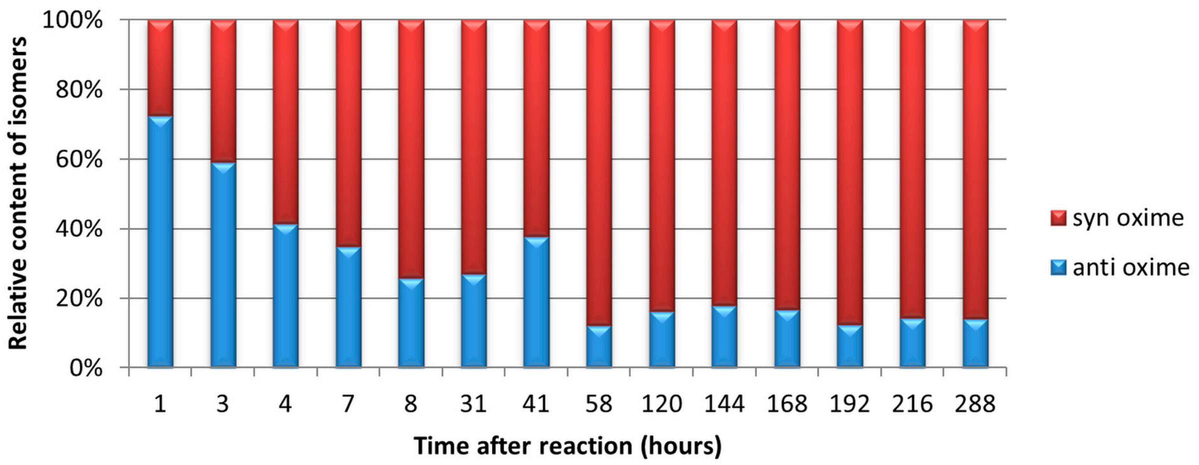

Fig. S5: Transformation of *anti*-1-methoxyindole-3-carbaledyhde oxime into *syn* isomer after milling without removal of excess reagents (5 eq. $NH_2OH.HCl$, 2 eq. NaOH, Pulverisette 6 mill, milling time 40 min).

It can be seen that one hour after reaction, the relative content of *anti* oxime was 72.5 % and its content decreased with time. The *syn* isomer became major after 4 hours. The majority of the conversion took place in the first 8 h after milling, when the relative content of *anti* oxime decreased to 25.9 %. A lower limit of 14 % *anti* isomer was established at 288 h (12 days).

4.2 Comparison of isomerisation and reaction progress in solution and in solid state

The kinetics of reaction/isomerization was investigated also for the aging experiment performed in WC chamber (Table 1, entry 9). In this case, also the situation after shorter time was investigated— the first three points correspond to 30, 60, and 90 min. Already in the first measurement, the content of aldehyde was below 25%. Further decrease was observed later and the values oscillated around 15% until the end of observation. The *anti*-*syn* isomerization also took place and the concentration of the *syn* isomer after 5 days was 63%. A significant increase in the amount of *syn* isomer was observed after washing procedure (up to 76%). It seems that if the system is activated for a very short time, the isomerization is slower and that the liquid medium facilitates it. The progress of the reaction and the *anti*-*syn* transformation are two processes that also continue after termination of milling.

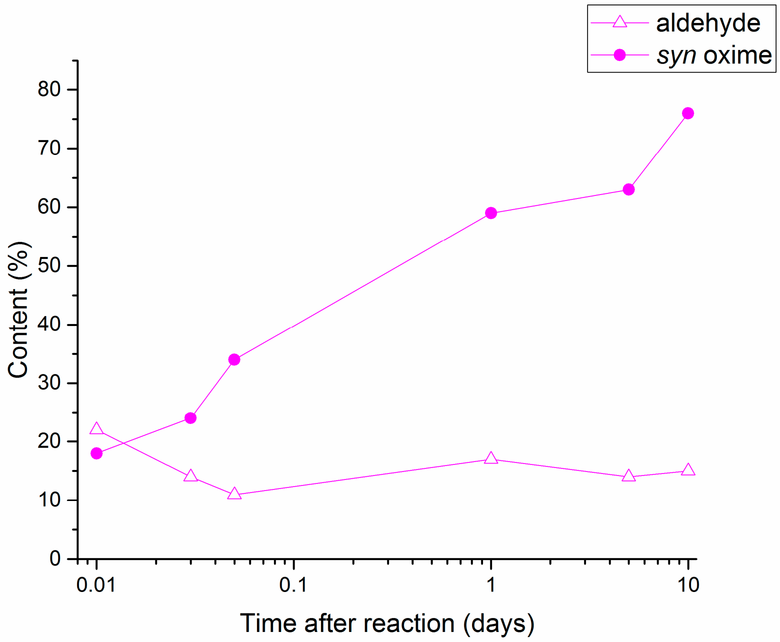

Fig. S6: Amounts of non-reacted aldehyde and syn oxime in the aging experiment performed in Pulverisette 7 premium line mill in WC chamber (Table 1, entry 9) determined from ^{1}H NMR spectra. Time of milling: 15 s. In this entry, 5 eq. $NH_2OH.HCl$ and 2 eq. NaOH were used. The data reported after 10 days are for the washed sample.

In addition to the solid-state transformation between the isomers and the reaction propagation in the presence of non-reacted hydroxylamine after the milling in solid state, these processes were also studied in a solvent by NMR measurements in DMSO. The NMR spectra were recorded 24 hours after dissolution and the amount of non-reacted aldehyde, as well as fractions of each oxime isomers were evaluated. The results are presented in Table S3, in which they are compared with the results obtained for the corresponding fresh powder.

Table S3: The amounts of non-reacted aldehyde and *anti* and *syn* oximes in selected experiments determined from ^{1}H NMR spectra after standing for one day in DMSO and in solid state. In entries 3, 5 and 8, 5 eq. $NH_2OH.HCl$ and 2 eq. NaOH were used. In entry 7, 5.2 eq. $NH_2OH.HCl$ and 2.7 eq. Na_2CO_3 was used.

**Experiment** (entry no. in Table 1: mill/material of milling media, time of milling)	**Aldehyde content (%)**		***anti*-oxime content (%)**		***syn*-oxime content (%)**	
	**in solution**	in powder	**in solution**	in powder	**in solution**	in powder
entry 3: NaOH, P7/WC, 20 min	**0**	0	**11**	10	**89**	90
entry 5: NaOH, P6/agate, 30 min, LAG	**0**	1	**37**	30	**63**	69
entry 7: Na_2CO_3, P7/WC, 40 min	**0**	0	**56**	57	**44**	43
entry 8: NaOH, P6/agate, 5 min	**0**	7	**47**	18	**53**	75

There are no significant differences in the samples stored in solution versus those stored in the solid state for both reaction progress and isomerization, with the exception of the diffusion experiment performed in agate (Table 1, entry 8), as no aldehyde was detected after one-day of storage in DMSO, whereas in the corresponding powder sample there was 7% remaining. The presence of solvent clearly facilitates the reaction progress. On the other hand, in terms of *anti-syn* oxime transformation, this process is kinetically faster than reaction completion, as the amount of *anti* oxime in the powder sample is much lower compared to the corresponding sample dissolved in DMSO. This suggests that the transformation of *anti* to *syn* isomer is an energetically more favorable process than the completion of the reaction in the solid state. When the reaction is completed during the milling procedure (other experiments mentioned in this table), the rate of the *anti-syn* transformation in solution is similar to that in the solid state.

In general, it seems that the increased surface energies in the reaction mass powders produced during the milling process facilitates both reaction propagation and isomerisation, as it is clear that it has the effects on reaction propagation hours after the termination of milling. The general conclusion is that upon tuning experimental conditions, namely the amount of base and hydroxylamine, but also milling conditions, it is possible to obtain almost pure *syn* isomer.

4.3 Isomerizaton of pure isomers

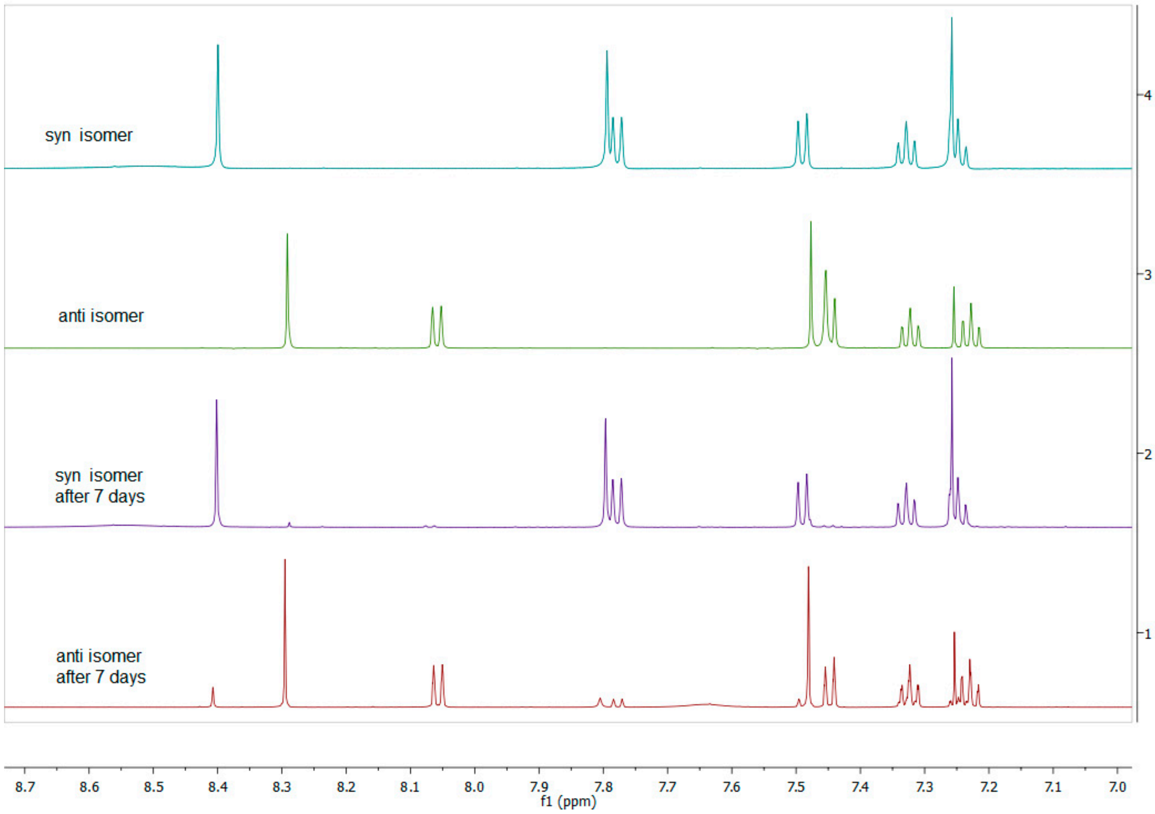

Fig. S7: Comparison of ^{1}H NMR spectra of pure *syn* and *anti* isomers after 7 days storage in $CDCl_3$ solution.

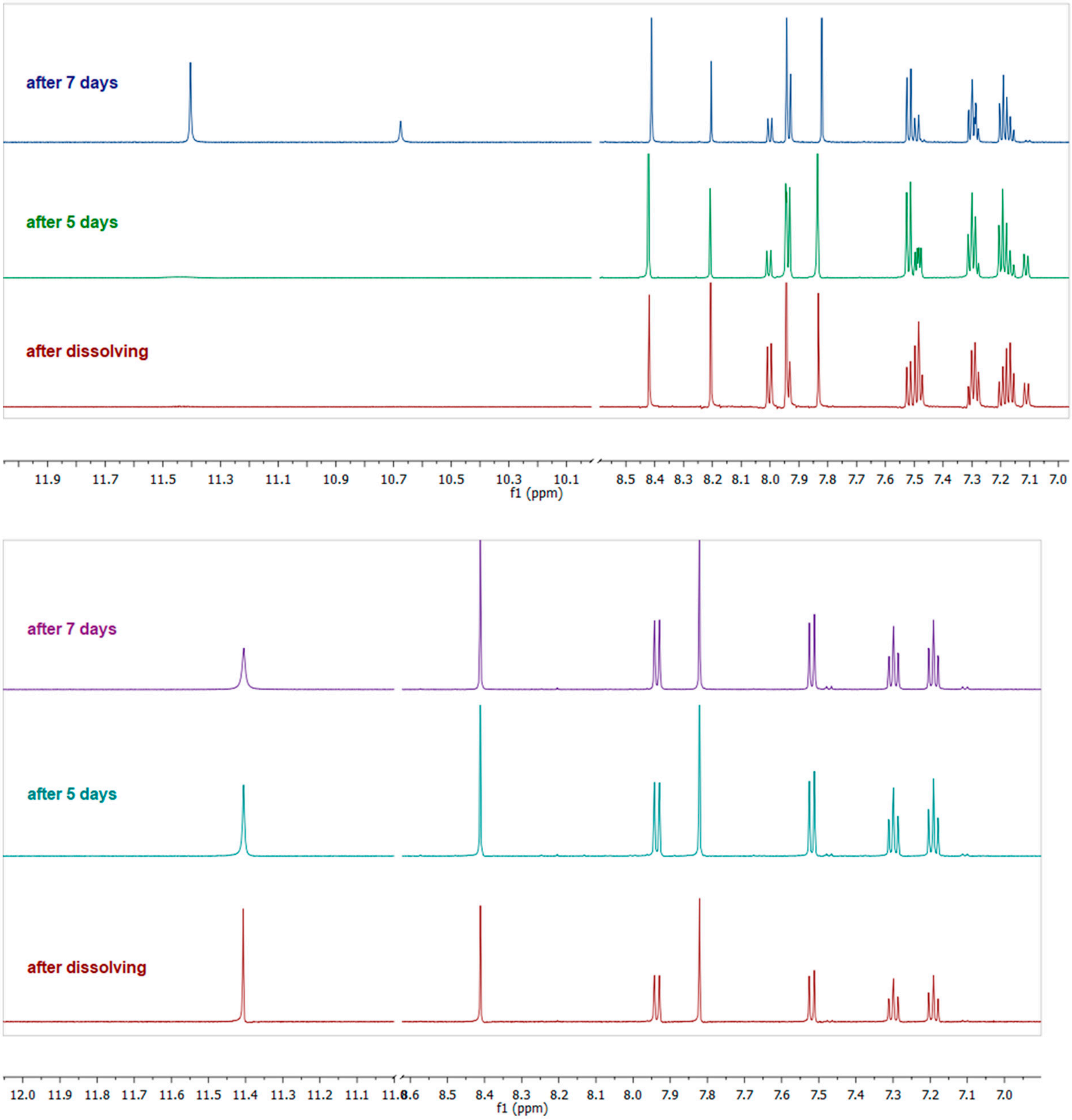

Fig. S8: Isomerisation of *anti* (top) and *syn* (bottom) oxime after milling (milling conditions: Pulverisette 7 mill, milling material WC, time 20 min) in the presence of 10% pTSA in solid state.

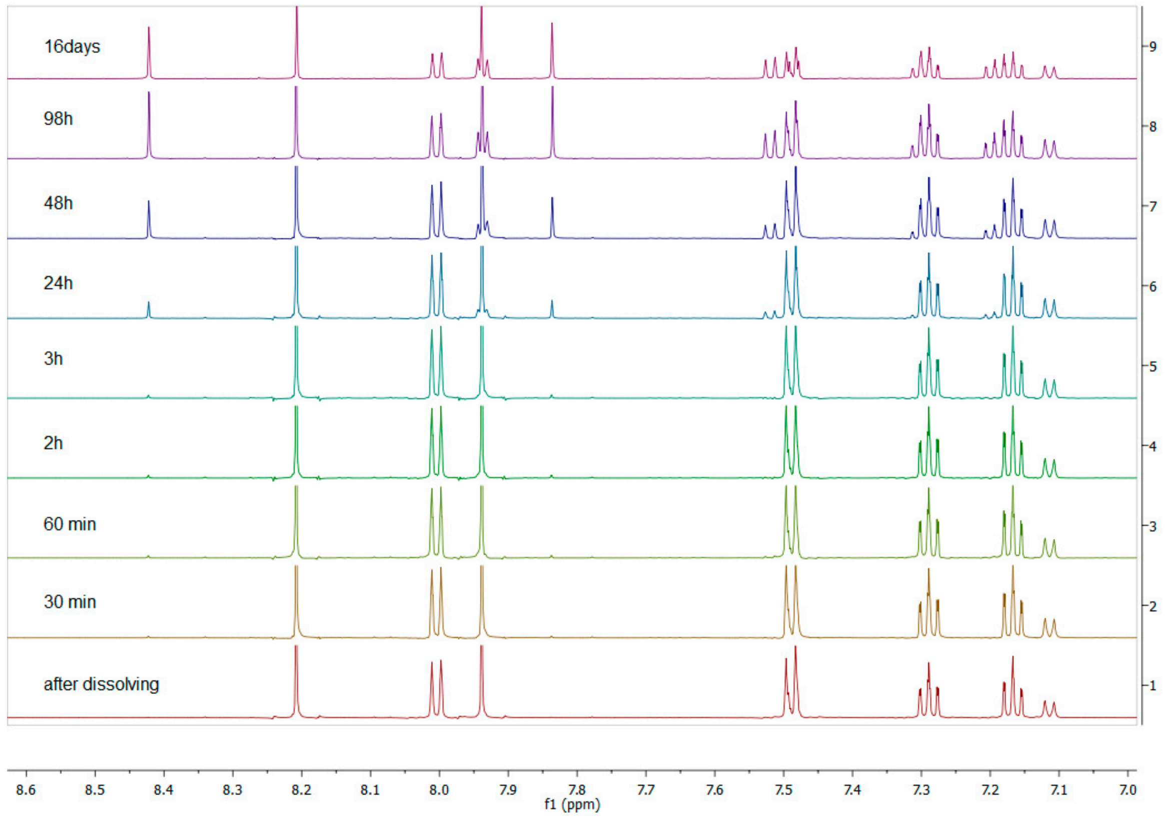

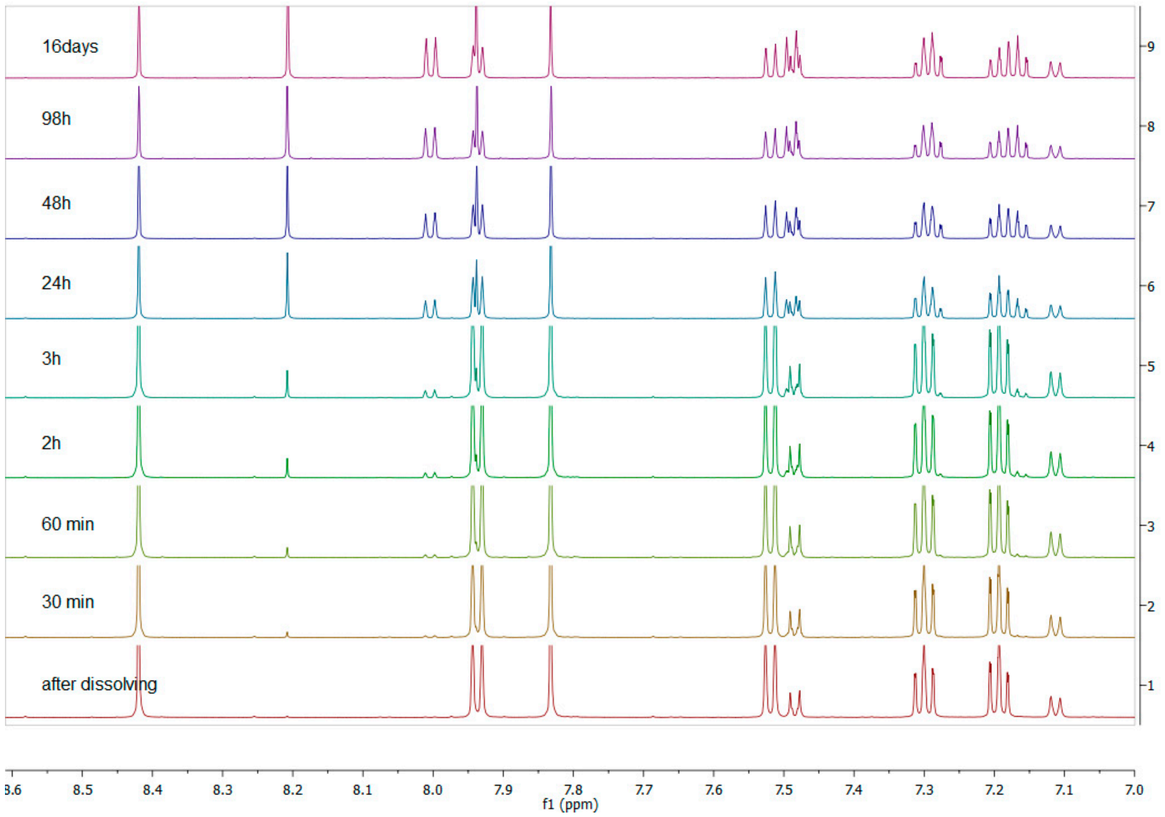

Fig. S9: Isomerisation of pure anti (top) and syn (bottom) oxime in the presence of 10% pTSA in DMSO.

5. Characterization of the products

5.1 NMR spectra of the starting materials and products

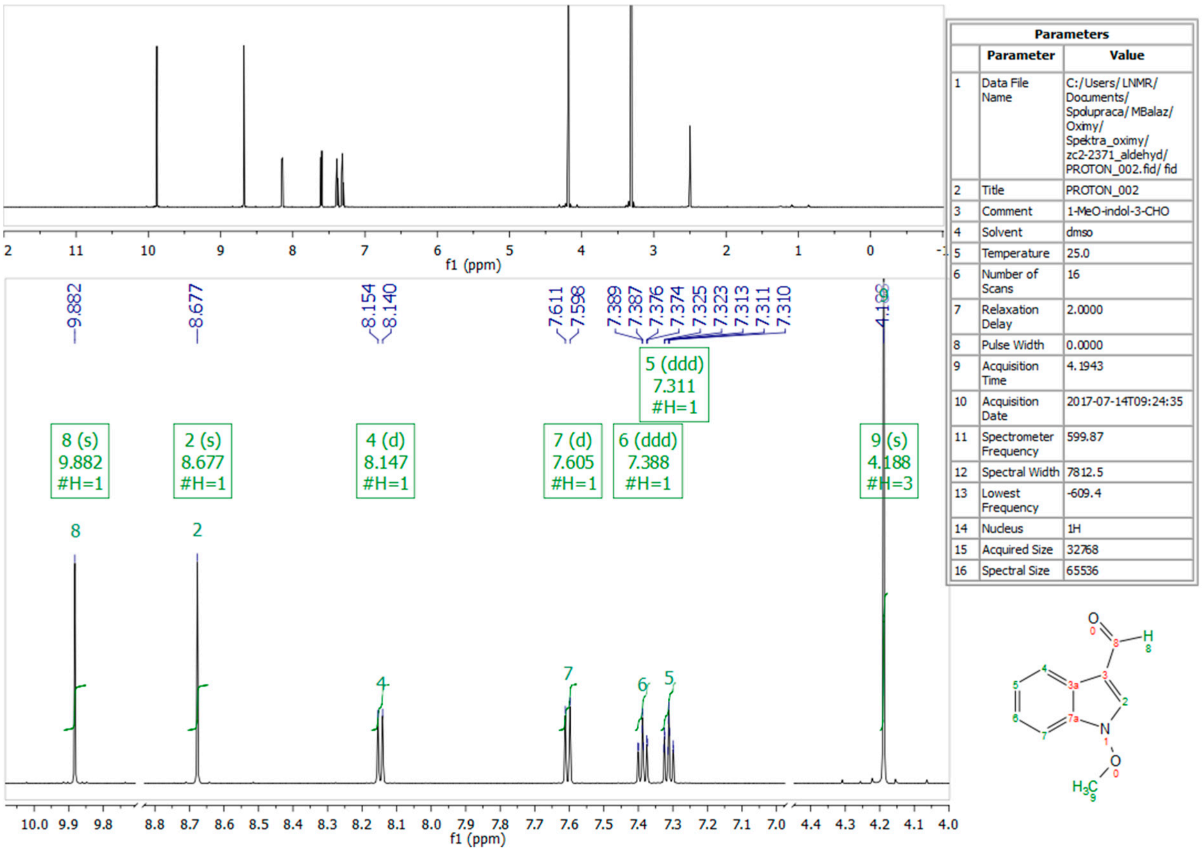

Parameters

	Parameter	Value
1	Data File Name	C:/Users/LNMR/Documents/Spolupraca/MBalaz/Oximy/Spektra_oximy/zc2-2371_aldehyd/PROTON_002.fid/fid
2	Title	PROTON_002
3	Comment	1-MeO-indol-3-CHO
4	Solvent	dmso
5	Temperature	25.0
6	Number of Scans	16
7	Relaxation Delay	2.0000
8	Pulse Width	0.0000
9	Acquisition Time	4.1943
10	Acquisition Date	2017-07-14T09:24:35
11	Spectrometer Frequency	599.87
12	Spectral Width	7812.5
13	Lowest Frequency	-609.4
14	Nucleus	1H
15	Acquired Size	32768
16	Spectral Size	65536

Fig. S10: ^{1}H NMR spectrum of starting 1-methoxyindole-3-carboxaldehyde 1 in DMSO

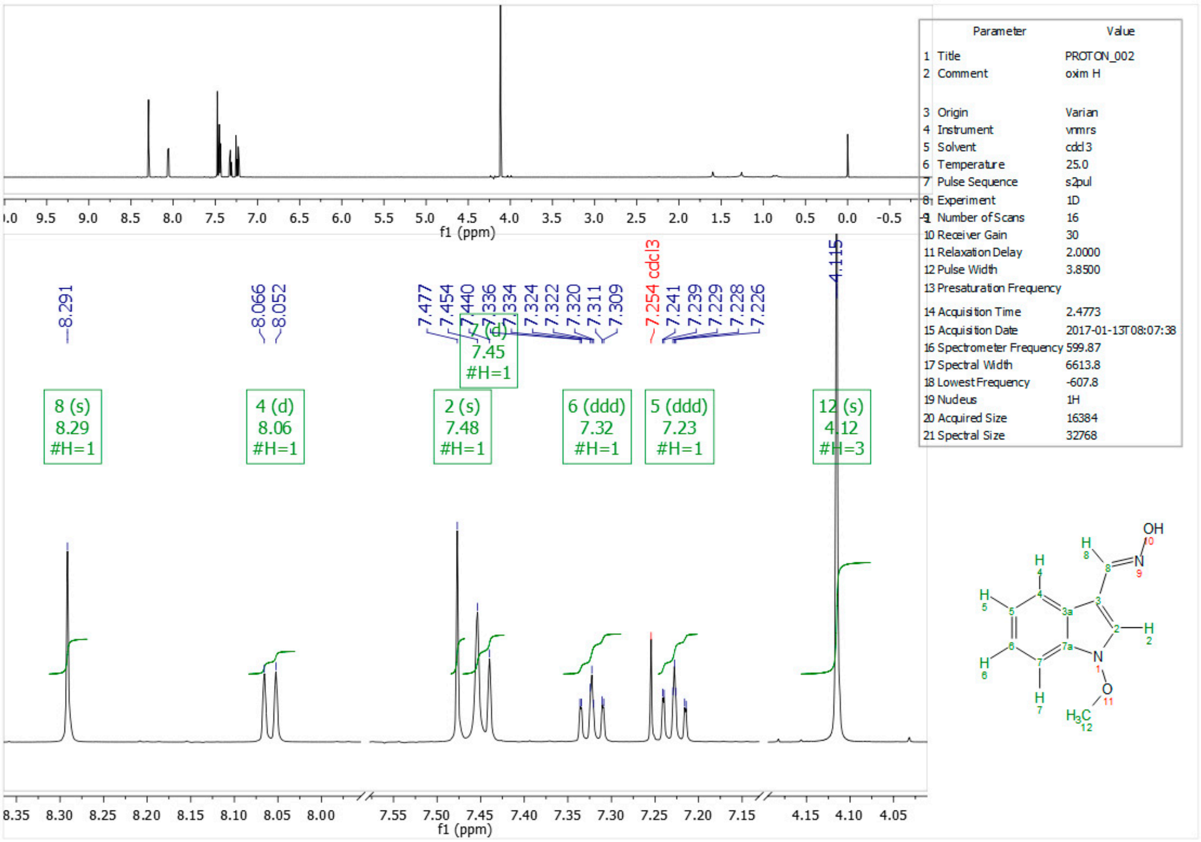

Fig. S11: ^{1}H NMR spectrum of *anti* 1-methoxyindole-3-carboxaldehyde oxime 1a in $CDCl_3$

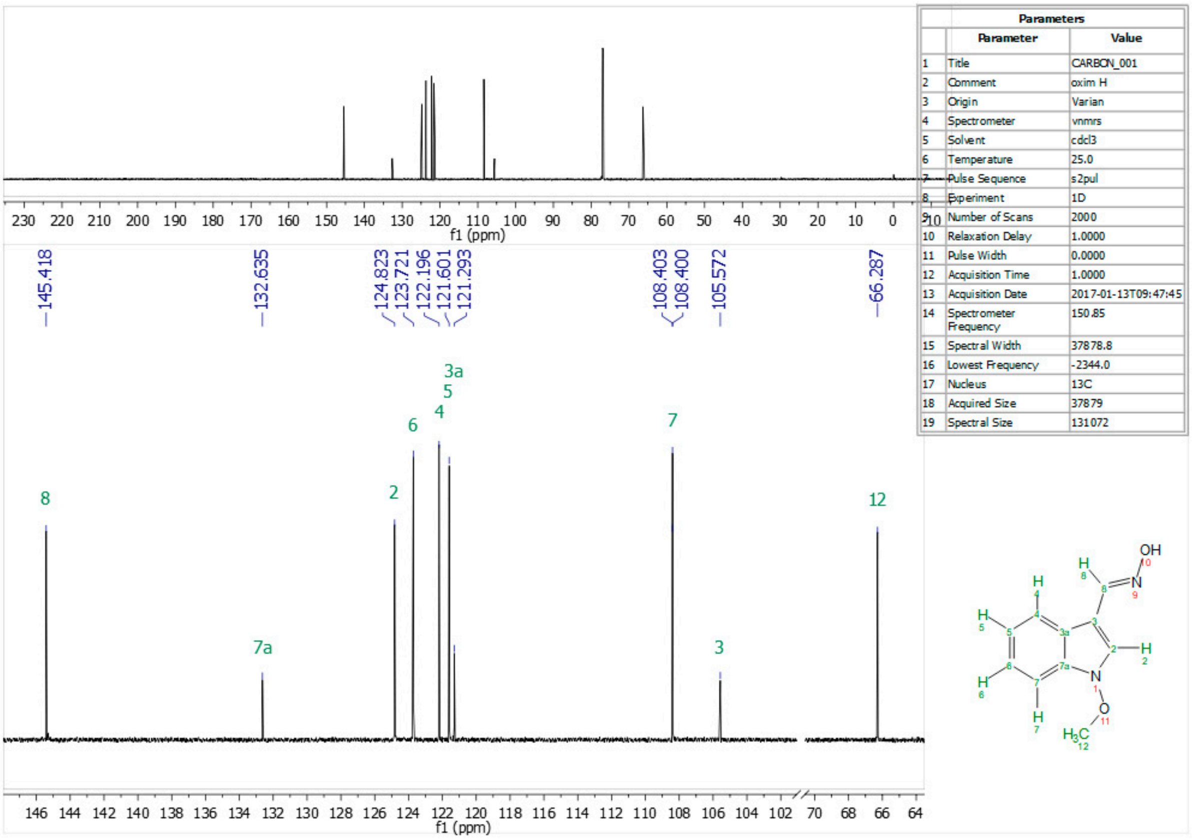

Fig. S12: ^{13}C NMR spectrum of *anti* 1-methoxyindole-3-carboxaldehyde oxime 1a in $CDCl_3$

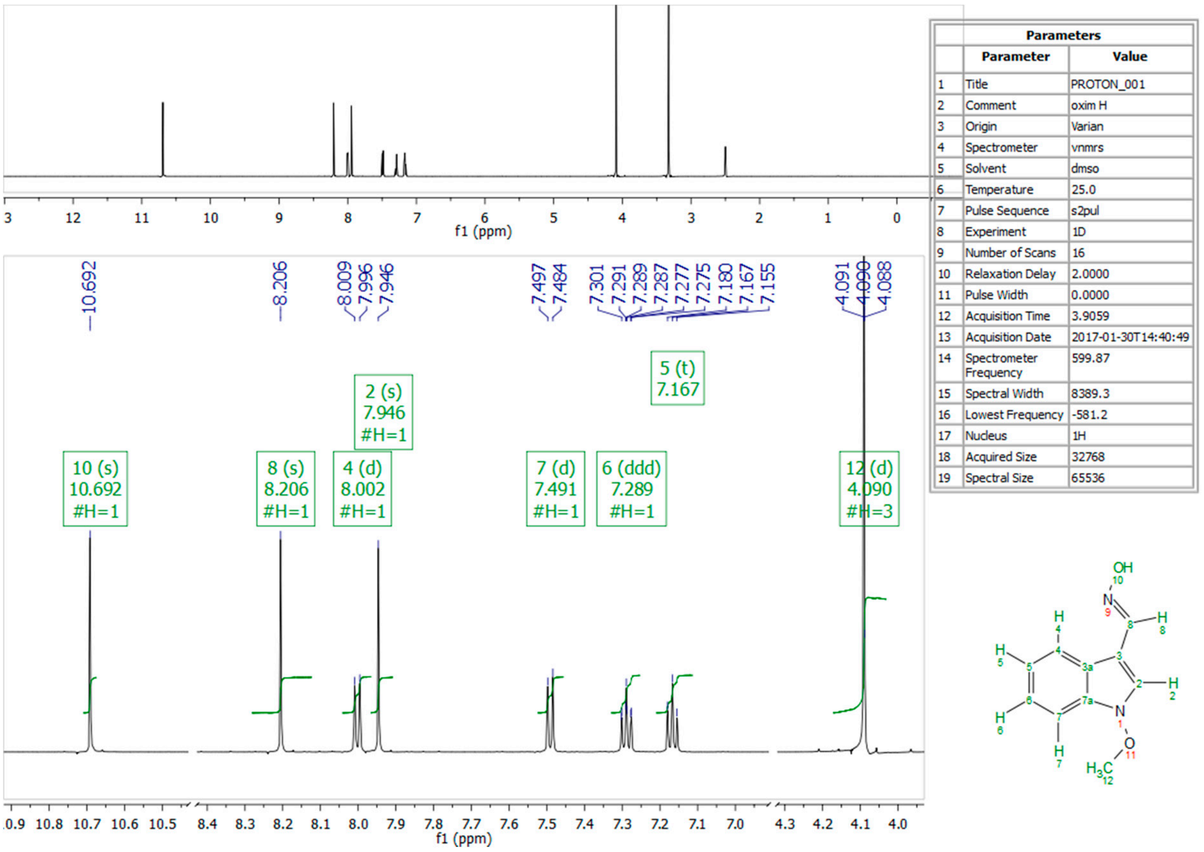

	Parameters	
	Parameter	Value
1	Title	PROTON_001
2	Comment	oxim H
3	Origin	Varian
4	Spectrometer	vnmrs
5	Solvent	dmso
6	Temperature	25.0
7	Pulse Sequence	s2pul
8	Experiment	1D
9	Number of Scans	16
10	Relaxation Delay	2.0000
11	Pulse Width	0.0000
12	Acquisition Time	3.9059
13	Acquisition Date	2017-01-30T14:40:49
14	Spectrometer Frequency	599.87
15	Spectral Width	8389.3
16	Lowest Frequency	-581.2
17	Nucleus	1H
18	Acquired Size	32768
19	Spectral Size	65536

Fig. S13: ¹H NMR spectrum of *anti* 1-methoxyindole-3-carboxaldehyde oxime 1a in DMSO

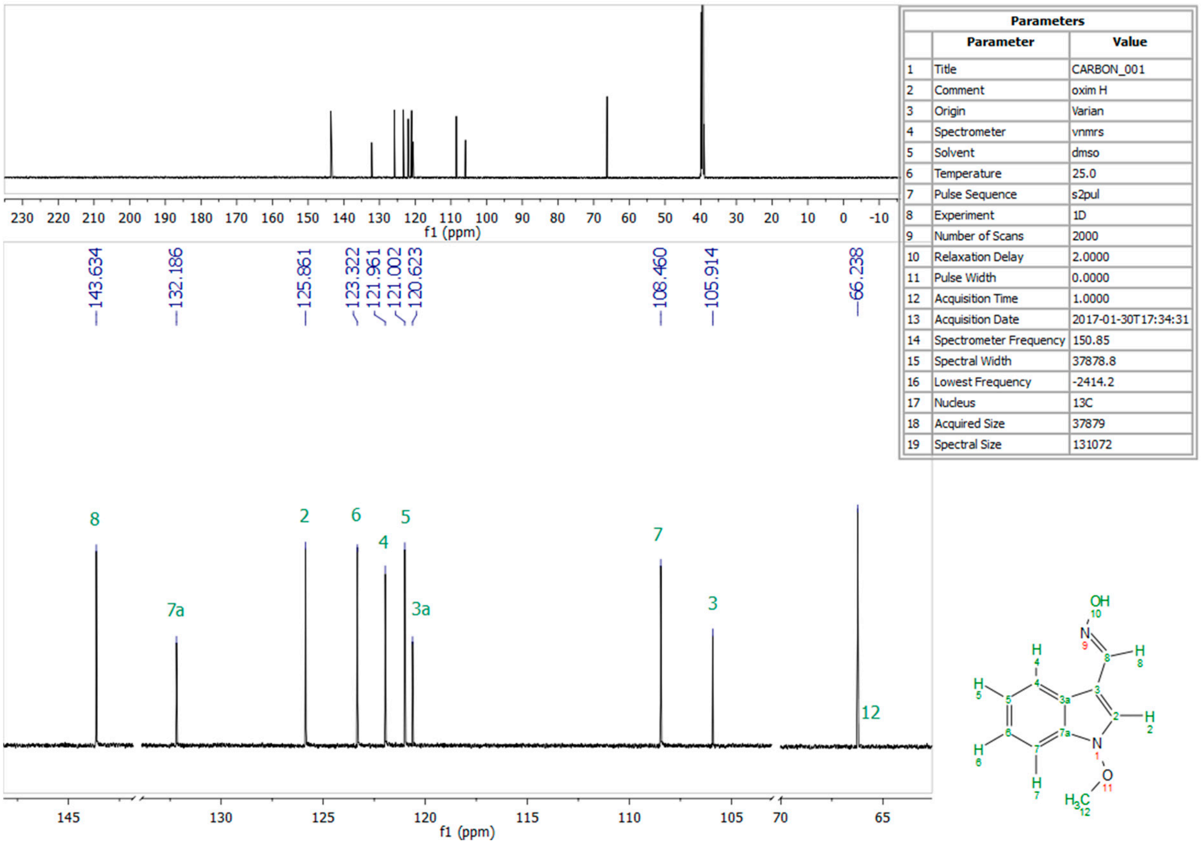

	Parameter	Value
1	Title	CARBON_001
2	Comment	oxim H
3	Origin	Varian
4	Spectrometer	vnmrs
5	Solvent	dmso
6	Temperature	25.0
7	Pulse Sequence	s2pul
8	Experiment	1D
9	Number of Scans	2000
10	Relaxation Delay	2.0000
11	Pulse Width	0.0000
12	Acquisition Time	1.0000
13	Acquisition Date	2017-01-30T17:34:31
14	Spectrometer Frequency	150.85
15	Spectral Width	37878.8
16	Lowest Frequency	-2414.2
17	Nucleus	13C
18	Acquired Size	37879
19	Spectral Size	131072

Fig. S14: ^{13}C NMR spectrum of *anti* 1-methoxyindole-3-carboxaldehyde oxime 1a in DMSO

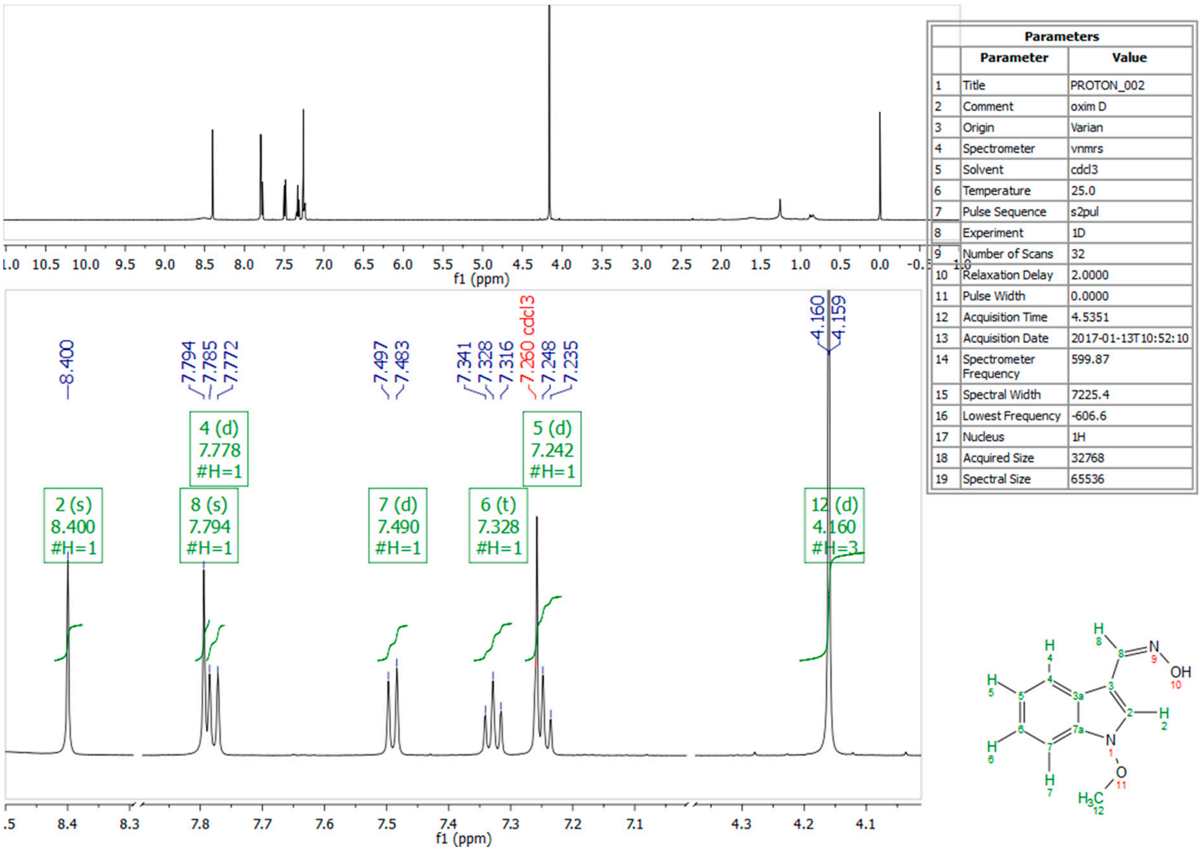

Parameters		
	Parameter	Value
1	Title	PROTON_002
2	Comment	oxim D
3	Origin	Varian
4	Spectrometer	vnmrs
5	Solvent	cdcl3
6	Temperature	25.0
7	Pulse Sequence	s2pul
8	Experiment	1D
9	Number of Scans	32
10	Relaxation Delay	2.0000
11	Pulse Width	0.0000
12	Acquisition Time	4.5351
13	Acquisition Date	2017-01-13T10:52:10
14	Spectrometer Frequency	599.87
15	Spectral Width	7225.4
16	Lowest Frequency	-606.6
17	Nucleus	1H
18	Acquired Size	32768
19	Spectral Size	65536

Fig. S15: ^{1}H NMR spectrum of *syn* 1-methoxyindole-3-carboxaldehyde oxime in $CDCl_3$

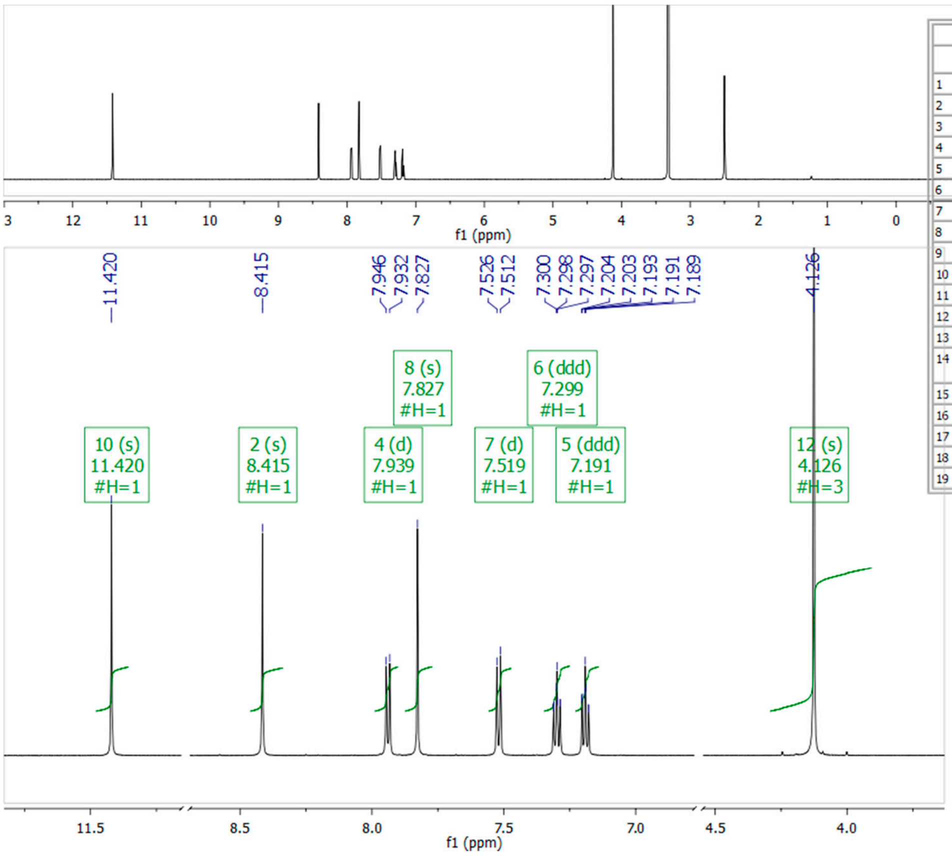

Parameters		
	Parameter	Value
1	Title	PROTON_002
2	Comment	oxim D
3	Origin	Varian
4	Spectrometer	vnmrs
5	Solvent	dmso
6	Temperature	25.0
7	Pulse Sequence	s2pul
8	Experiment	1D
9	Number of Scans	16
10	Relaxation Delay	2.0000
11	Pulse Width	0.0000
12	Acquisition Time	3.9059
13	Acquisition Date	2017-01-31T11:40:50
14	Spectrometer Frequency	599.87
15	Spectral Width	8389.3
16	Lowest Frequency	-584.3
17	Nucleus	1H
18	Acquired Size	32768
19	Spectral Size	65536

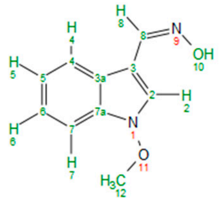

Fig. S16: ^{1}H NMR spectrum of *syn* 1-methoxyindole-3-carboxaldehyde oxime 1a in DMSO

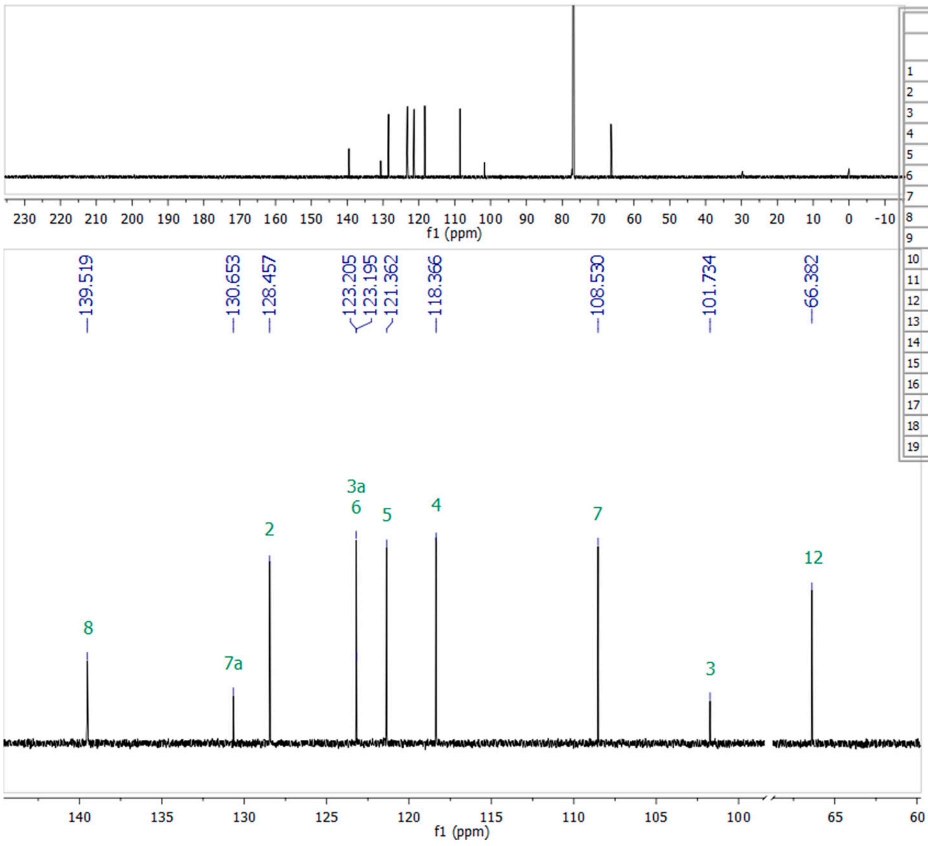

Parameters		
	Parameter	Value
1	Title	CARBON_001
2	Comment	oxim D
3	Origin	Varian
4	Spectrometer	vnmrs
5	Solvent	cdcl3
6	Temperature	25.0
7	Pulse Sequence	s2pul
8	Experiment	1D
9	Number of Scans	2000
10	Relaxation Delay	1.0000
11	Pulse Width	0.0000
12	Acquisition Time	1.0000
13	Acquisition Date	2017-01-13T12:32:15
14	Spectrometer Frequency	150.85
15	Spectral Width	37878.8
16	Lowest Frequency	-2343.1
17	Nucleus	13C
18	Acquired Size	37879
19	Spectral Size	131072

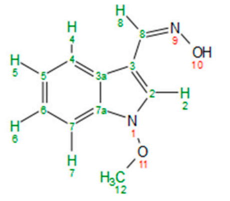

Fig. S17: ^{13}C NMR spectrum of *syn* 1-methoxyindole-3-carboxaldehyde oxime 1a in $CDCl_3$

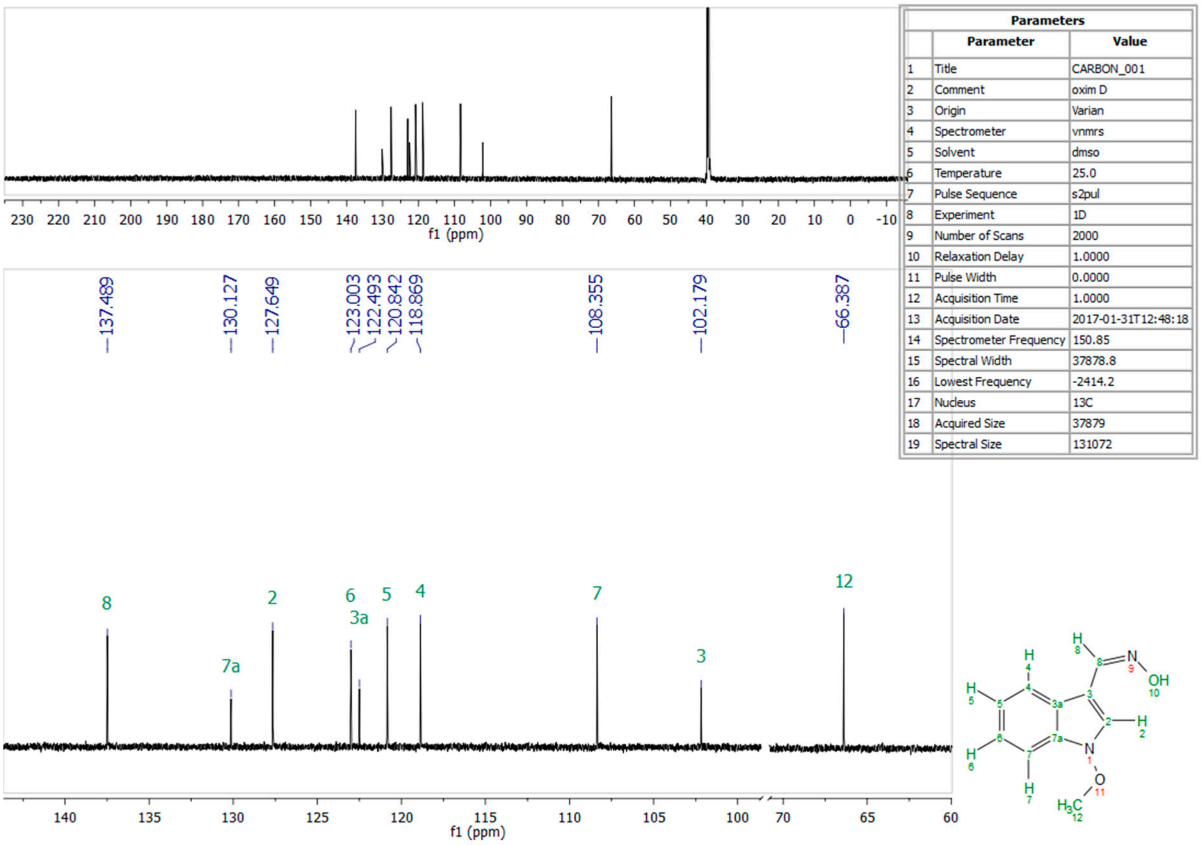

Parameters		
	Parameter	Value
1	Title	CARBON_001
2	Comment	oxim D
3	Origin	Varian
4	Spectrometer	vnmrs
5	Solvent	dmso
6	Temperature	25.0
7	Pulse Sequence	s2pul
8	Experiment	1D
9	Number of Scans	2000
10	Relaxation Delay	1.0000
11	Pulse Width	0.0000
12	Acquisition Time	1.0000
13	Acquisition Date	2017-01-31T12:48:18
14	Spectrometer Frequency	150.85
15	Spectral Width	37878.8
16	Lowest Frequency	-2414.2
17	Nucleus	13C
18	Acquired Size	37879
19	Spectral Size	131072

Fig. S18: ^{13}C NMR spectrum of *syn* 1-methoxyindole-3-carboxaldehyde oxime 1a in DMSO

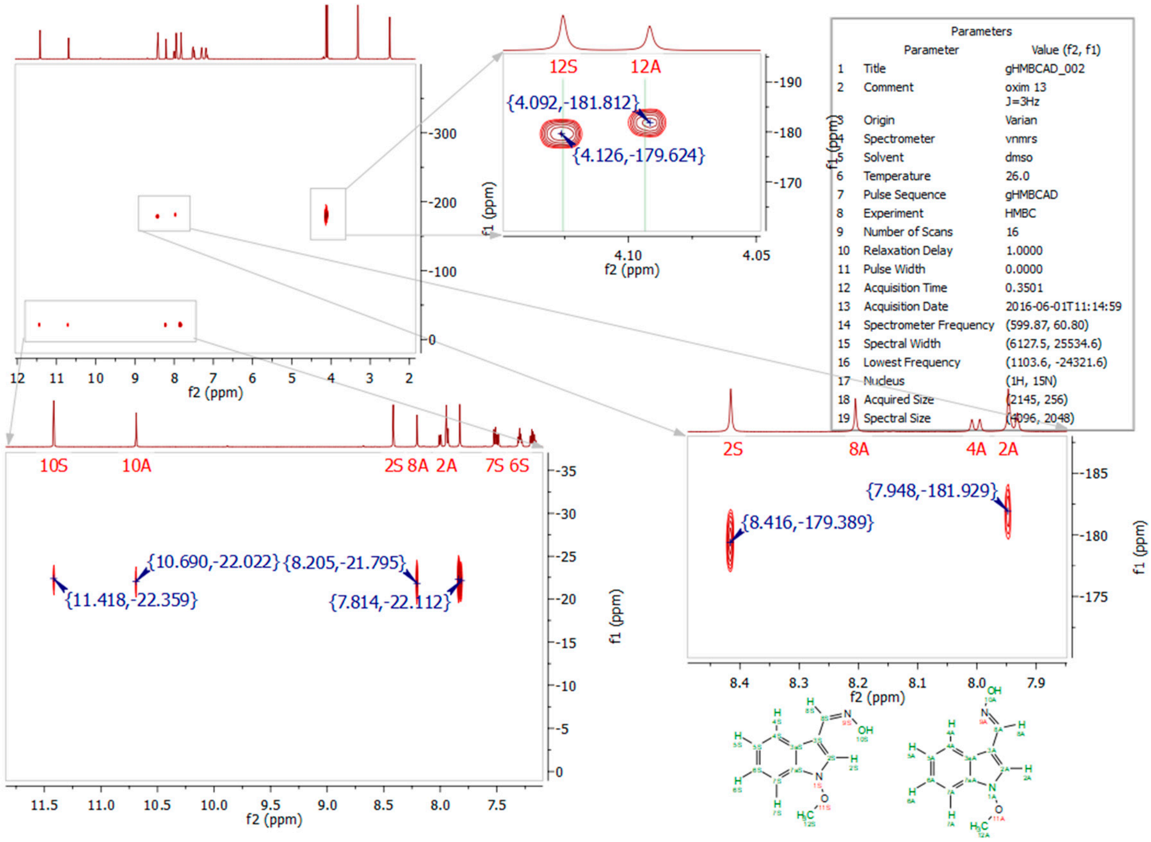

Fig. S19: ^{15}N HMBC NMR spectra of *syn* and *anti* 1-methoxyindole-3-carboxaldehyde oxime 1a

5.2 Characterization of anti and syn-1-methoxyindolecarboxaldehyde oximes 1a

Individual isomers of the product after performing the synthesis under ideal conditions (in particular Pulverisette 7 mill, WC milling material, 20 minutes of treatment, 500 rpm milling speed and ball-to-powder ratio 71 and the same amount of both hydroxylamine and base, i.e. Table 1, entry 4) were obtained by chromatographic separation. The compounds were isolated with yields 31% and 56% for *anti* and *syn* isomer, respectively. Although the *anti* isomer was major before the column chromatography separation, the *anti*-*syn* isomerization occurred also during the process resulting in higher yield of the *syn* isomer.

5.2.1 Nuclear magnetic resonance measurements

The pure oxime isomers were analyzed by NMR spectroscopy. The ^{1}H, ^{13}C, and ^{15}N chemical shifts and coupling constants of both isomers are given in Table S4-6.

Table S4: ^{1}H chemical shifts (ppm) in the *syn* and *anti* isomers 1a (in DMSO-d$_6$)

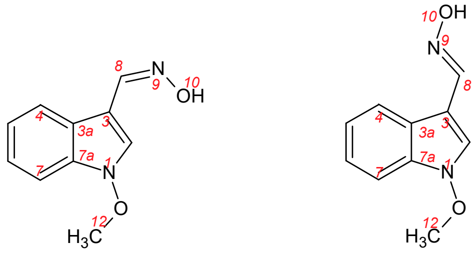

syn (*Z*) *anti* (*E*)

Cmpd.	**H-2**	**H-4**	**H-5**	**H-6**	**H-7**	**H-8**	**H-10**	**H-12**
syn (*Z*)	8.42	7.94	7.20	7.30	7.52	7.83	11.42	4.13
anti (*E*)	7.95	8.00	7.17	7.29	7.49	8.21	10.69	4.09
syn - anti	+0.47	-0.06	+0.03	+0.01	+0.03	-0.38	+0.73	+0.04

Table S5: ^{13}C and ^{15}N chemical shifts (ppm) in the *syn* and *anti* isomers 1a (in DMSO-d$_6$)

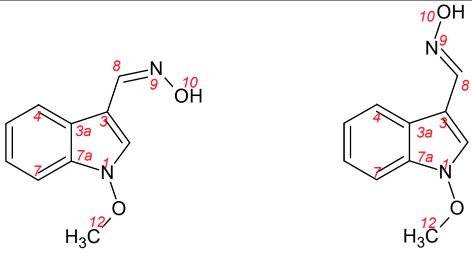

syn (*Z*) *anti* (*E*)

Cmpd.	**C-2**	**C-3**	**C-4**	**C-5**	**C-6**	**C-7**	**C-8**	**C-3a**	**C-7a**	**C-12**	**N-1**	**N-9**
syn (*Z*)	127.6	102.2	118.9	120.8	123.0	108.4	137.5	122.5	130.1	66.4	-179.4	-22.1

anti (*E*)	125.9	105.9	122.0	121.0	123.3	108.5	143.6	120.6	132.2	66.2	-181.8	-21.8
syn - *anti*	+1.7	-3.7	-3.1	-0.2	-0.3	-0.1	-6.1	+1.9	-1.1	+0.2	+2.4	-0.3

Table S6: ^{13}C–^{1}H coupling constants (Hz) in the *syn* and *anti* isomers 1a (in DMSO-d_6)

Cmpd.	$^1J_{C2/H2}$	$^1J_{C4/H4}$	$^1J_{C5/H5}$	$^1J_{C6/H6}$	$^1J_{C7/H7}$	$^1J_{C8/H8}$
syn (*Z*)	195.6	154.8	159.6	156.6	163.8	172.2
anti (*E*)	189.6	162.6	158.4	154.8	164.4	161.4
syn - *anti*	+6.0	-7.8	+1.2	+1.8	-0.6	+10.8

The largest changes of the chemical shifts (*syn* - *anti*) were obtained for the indole H-2 (+0.47 ppm), aldoxime H-8 (-0.38 ppm) and OH (+0.73 ppm) protons (Table S4). ^{13}C chemical shifts changes (*syn* - *anti*) corresponded with the ^1H ones for carbons C-2 (+1.7 ppm) and C-8 (-6.1 ppm). Moreover, shielding effect was found for C-3 (-3.7 ppm) and C-4 (-3.1 ppm) signals, while the latter was not reflected for the attached H-4 proton (-0.06 ppm). The reason may be rather a resonance effect within the aromatic benzene ring of indole than hydrogen bonding of H-4 (Table S5).

The analysis of coupling constants and NOE enhancements allowed the elucidation of the spatial structure of the both stereoisomers. The $^1J_{C8/H8}$ coupling constant is greater by 10.8 Hz in the *syn* isomer than in the *anti* one, since the nitrogen lone pair occupies a *cis* position relative to C-8–H-8 bond [4]. In the *syn* isomer, the coupling constant $^1J_{C2/H2}$ increased by 6.0 Hz and $^1J_{C4/H4}$ decreased by -7.8 Hz. We assume that the hydrogen bond present between the hydroxyl oxygen and proton H-2 in the *syn* isomer may lead to an increase of the $^1J_{C2/H2}$ coupling constant in comparison with the *anti* isomer (Table S6). The spatial orientation of the aldoxime fragment with reference to the indole plane in both isomers can be defined on the basis of geminal $^2J_{C3/H8}$ and vicinal $^3J_{C2/H8}$ coupling constants too. The observed value 8.4 Hz of the vicinal coupling constant $^3J_{C2/H8}$ corresponds to mutual *trans* position of carbon C-2 and proton H-8 in the *syn* isomer and the vicinal coupling constant $^3J_{C2/H8}$ = 2.9 Hz corresponds to mutual *cis* position of carbon C-2 and proton H-8 in the *anti* isomer [5].

The *syn* structure was further corroborated by 1D selective NOESY measurements where spatial proximity of =CH-8 and indole H-4 protons had been unambiguously proved by great mutual NOE enhancements 6.20% and 6.35% (Fig. S20), unlike the *anti* structure having those enhancements minimal, only 0.62% and 0.67%. On the other hand, the spatial proximity of =CH-8 and indole H-2 protons in the *anti* structure was confirmed by much greater mutual NOE enhancements, 2.87% and 2.96%, whereas much larger distance between these protons in the *syn* structure can be seen from minimal NOE values 0.33% and 0.39%. Moreover, hydrogen bond formation between

hydroxyl oxygen and proton H-2 in the *syn* isomer may be anticipated because of favorable six-membered arrangement (Fig. S20).

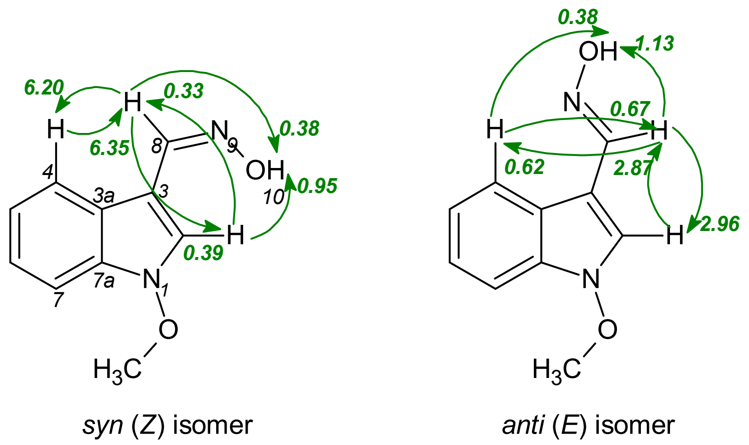

Fig. S20: Structures of the both isomers of 1a and NOESY enhancements found (measured in DMSO-d6)

5.2.2 X-ray diffractometry

In order to identify the most important diffractions of both isomers, XRD patterns were recorded (Fig. S21).

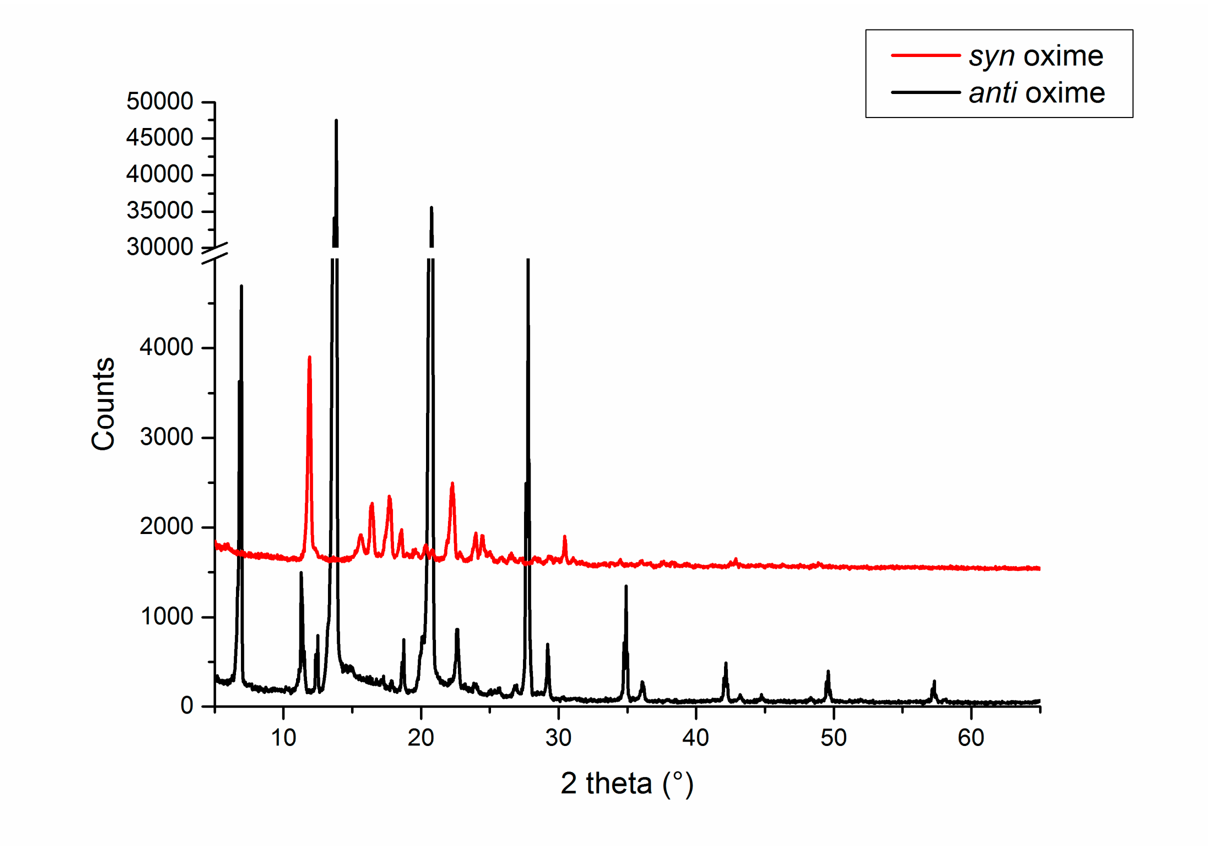

Fig. S21: XRD patterns of pure oximes 1a: *syn* (red) and *anti* (black).

As it was expected, the pure isomers exhibit very different XRD patterns. The relative intensity of the *anti* oxime is much higher than that of the *syn* oxime, thus indicating significantly larger crystals in the former case. The maximum intensities of the most intensive peaks located at 13.8° and 20.8° were 47469 and 35572 counts, respectively (the intensity maxima are not shown in the figure, as they are too high). The approximate crystallite sizes of the *syn* and *anti* isomer were 42 and 75 nm, respectively.

5.2.3 FT-infrared spectra

The infrared spectra of the pure oxime isomers are provided in (Fig. S22).

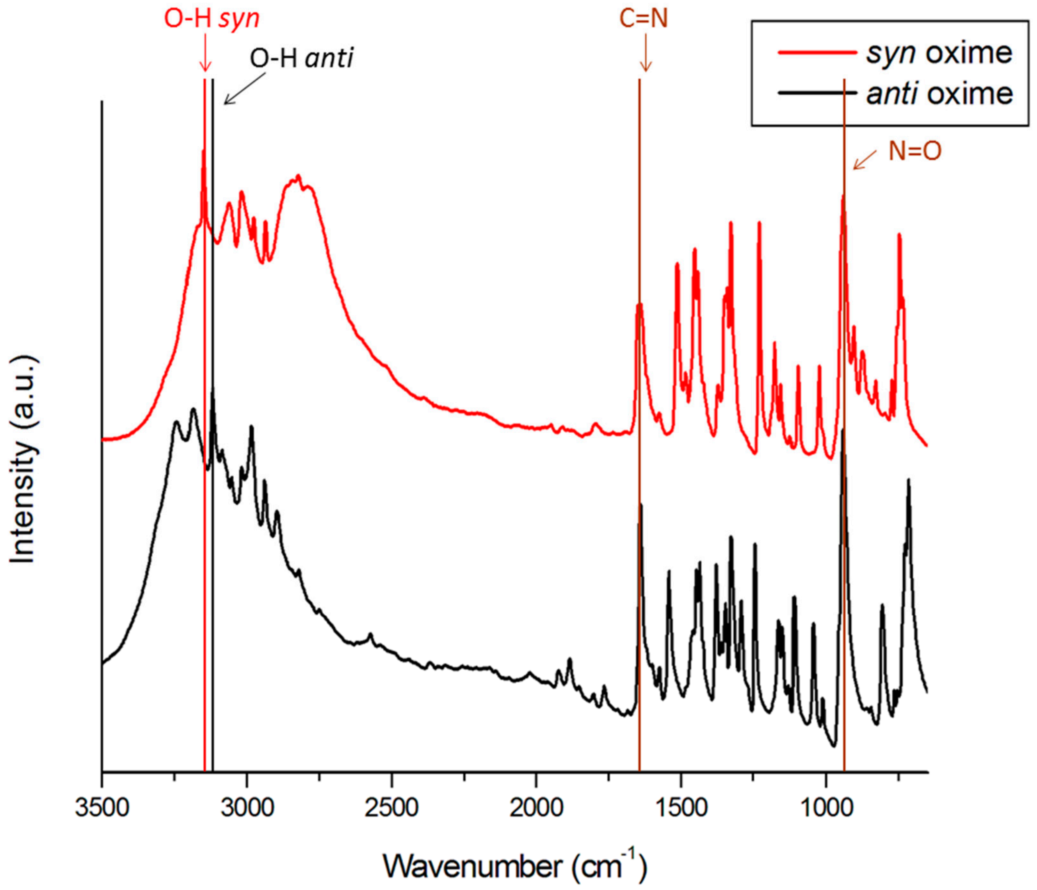

Fig. S22: Infrared spectra of pure oximes 1a.

The spectra of both isomers are different, similarly as in the case of XRD. The corresponding bands, namely those belonging to oxime functional group are important. In the case of *syn* oxime, we have observed the vibrations of O-H, C=N and N=O bonds at 3150, 1639 and 941 cm^{-1}, respectively. In the case of *anti* oxime, the vibrations of C=N and N=O groups were similar, however, the O-H bond vibration was present at 3120 cm^{-1}. The wavenumbers of these bonds are in accordance with the values reported in literature [6].

5.2.4 Analysis of melting point temperature

The pure isomers had melting points of 93-95 °C for *anti* and 137-139 °C for *syn* oxime. The former temperature range is in accordance with 94-96 °C reported by Hanley et al. [3], who, according to the reported NMR shifts, have isolated the pure *anti* isomer. The obtained values are quite similar as in ref. [7] (98.5-99.5 °C and 146-147 °C for *syn* and *anti*, respectively), however, it seems that the values are reported vice versa in that study.

5.2.5 Optical microscopy

Both pure isomers were analyzed by optical microscopy. The results are provided in Fig. S23 below.

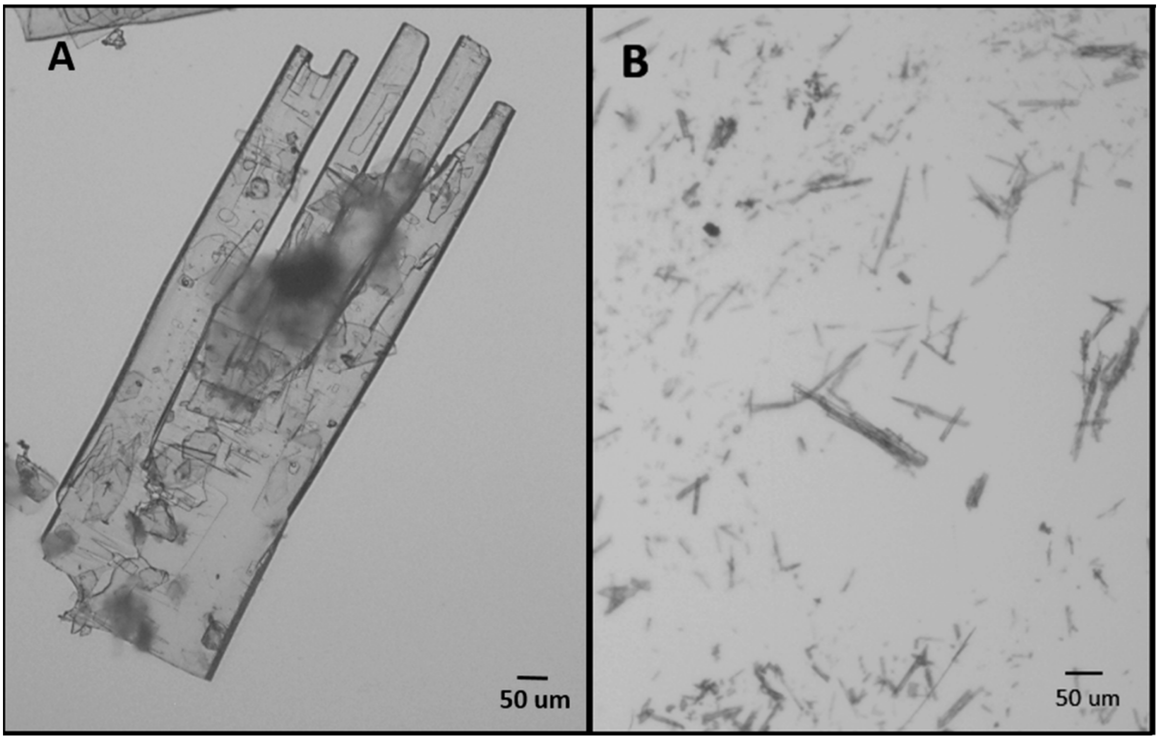

Fig. S23: Photographs of the oxime 1a isomers crystals from the optical microscope: (A) *anti*; (B) *syn*.

Large differences in the crystallite size between both the oxime isomers can be observed. The differences concern mainly the length and width of the crystals. In general, the crystals of the *anti* isomer are much larger, as can be seen from Fig. S26, in which the same magnification is used. A large crystal of the *anti* isomer shown in Fig. S23a is 937 μm long (maximum) and 306 μm wide. It seems like smaller crystallites are growing out of one common crystal. The size of the crystals of the *anti* isomers is very heterogeneous, ranging from 300 μm to 1500 μm in length and from 35 μm to 350 μm in width. The average length of the crystals of the *anti* isomer is 527 μm and their average width is 115 μm. On the other hand, the length of the observed crystals of *syn* isomer (Fig. S22b) is from 20 to 90 μm (average value 45 μm) and the average width is 11 μm, so the overall size is much smaller. The heterogeneity of the morphology of the *syn* isomer seems to be much smaller. The morphology of both isomers is rod-shaped.

The different morphology of the two isomers is visible also without the utilization of the microscope, as the crystals of the *anti* isomer gleam and the *syn* ones remind paper (Fig. S24). When the same quantity of both isomers is weighed, the *syn* ones occupy much more space.

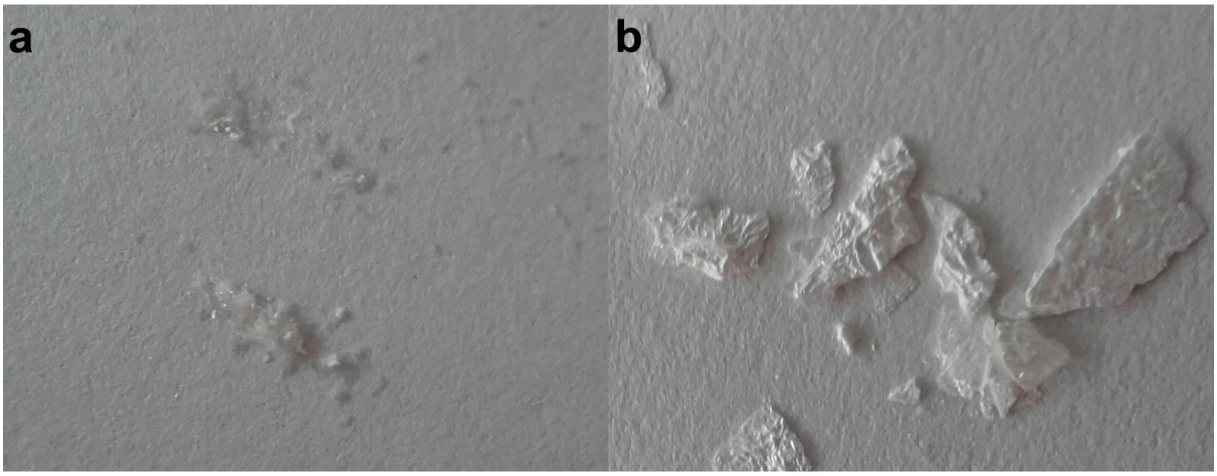

Fig. S24: Photographs of the crystals of the isolated isomers of 1-methoxycarboxyaldehdyde oxime 1a: (a) *anti* ; (b) *syn*.

5.2.6 Transmission electron microscopy

TEM analyses were performed in order to observe the morphology of oxime isomers in more detail. Typical low-magnification TEM images of both isomers are shown in Fig. S25. The morphology of particles in both samples can be described as two-dimensional. The particles of both isomers occur in the form of very thin electrons transparent sheets, indicating that their thickness is in the range of the supporting lacey carbon film, which is around 10 nm. The average size of the *syn* oxime fragments (Fig. S25a) on the TEM grid in the other two dimensions is in the micron range, whereas in the *anti* oxime sample (Fig.S25b), they extend over several microns. This supports the results from the optical microscopy. Bending of sheets at the edges is regularly observed in both samples. EDS analyses have confirmed the presence of C (also from the carbon-coating), N and O in both samples. The presence of small amount of Si was detected as a result from the agate milling media, despite the fact that the samples were purified by column chromatography. Due to the extremely small thickness and high sensitivity of the organic samples to high-energy electrons, crystallinity of the samples was not confirmed in the TEM.

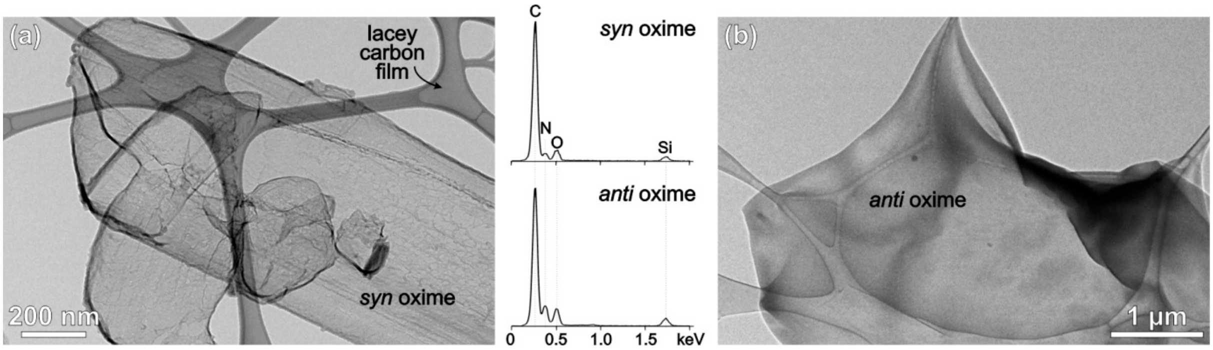

Fig. S25: Typical low-magnification images of the (a) *syn* oxime 1a and (b) *anti* oxime 1a samples revealing their 2-dimensional morphology. EDS spectra of both samples with the nitrogen peak, characteristic for both oxime isomers are provided in the middle.

6. Substrate scope in the oximation-isomerization reactions

In order to investigate if the proposed methodology is applicable also for other N-substituted indole-3-carboxaldehydes oximations, the experiments with 2 other substituents and non-substituted analogue were performed (Table 2 in the main body of the paper). The NMR data for the products are summarized here. For each analog, the ^{1}H and ^{13}C NMR spectrum from the complex figure with assigned peaks for *syn*/*anti* isomers is then analyzed in detail in the figures below the complex one.

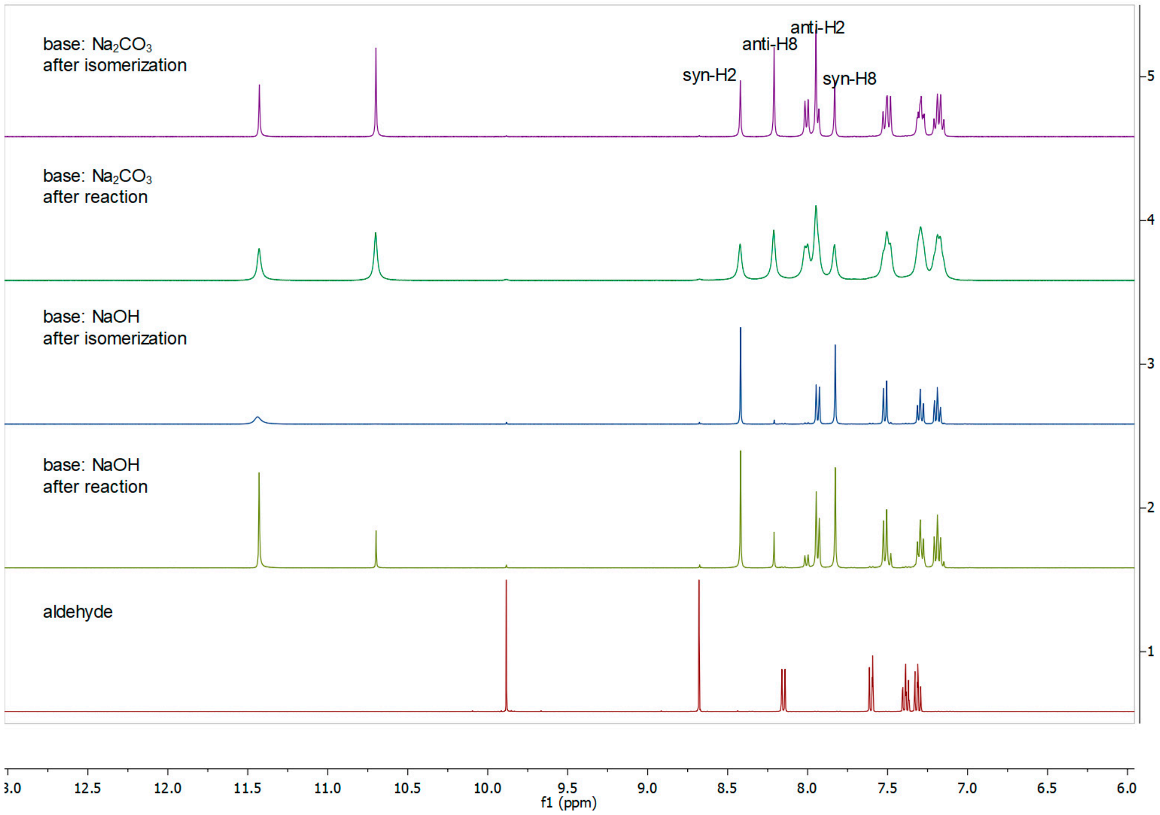

Fig. S26: NMR spectra of the products of the reaction of 1-methoxyindol-3-carboxaldehyde 1 with $NH_2OH.HCl$ and a base (indicated in figure) recorded after reaction and after isomerisation.

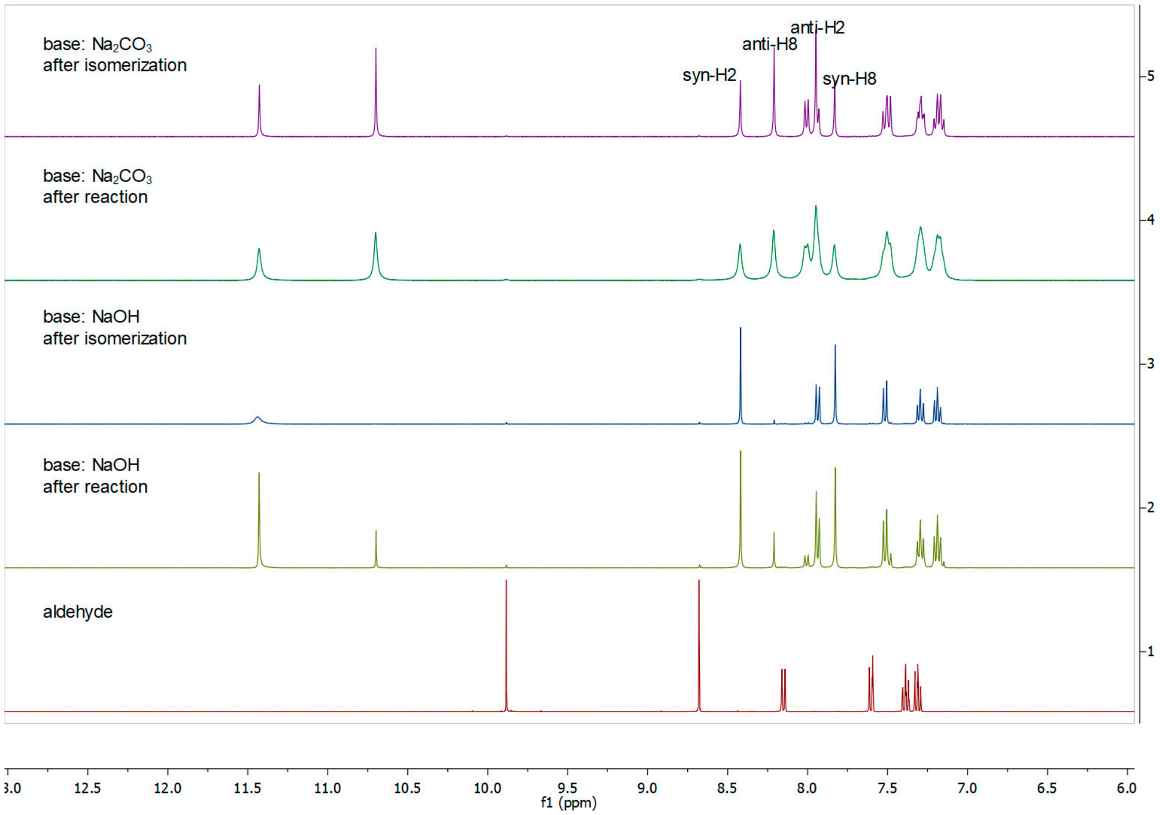

Fig. S27: NMR spectra of the products of the reaction of indol-3-carboxaldehyde 2 with $NH_2OH.HCl$ and a base (indicated in figure) recorded after reaction and after isomerisation.

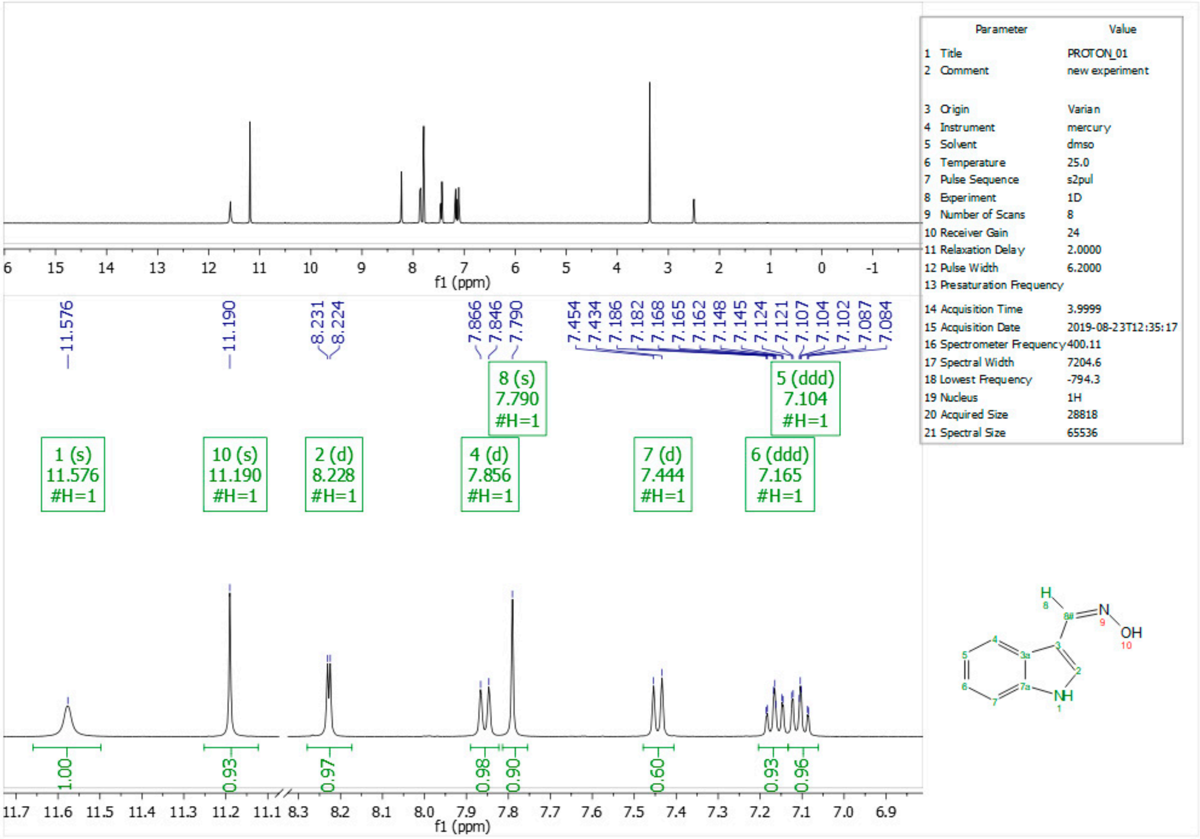

Fig. S28: ¹H NMR spectrum of *syn* indole-3-carboxaldehyde oxime 2a in DMSO.

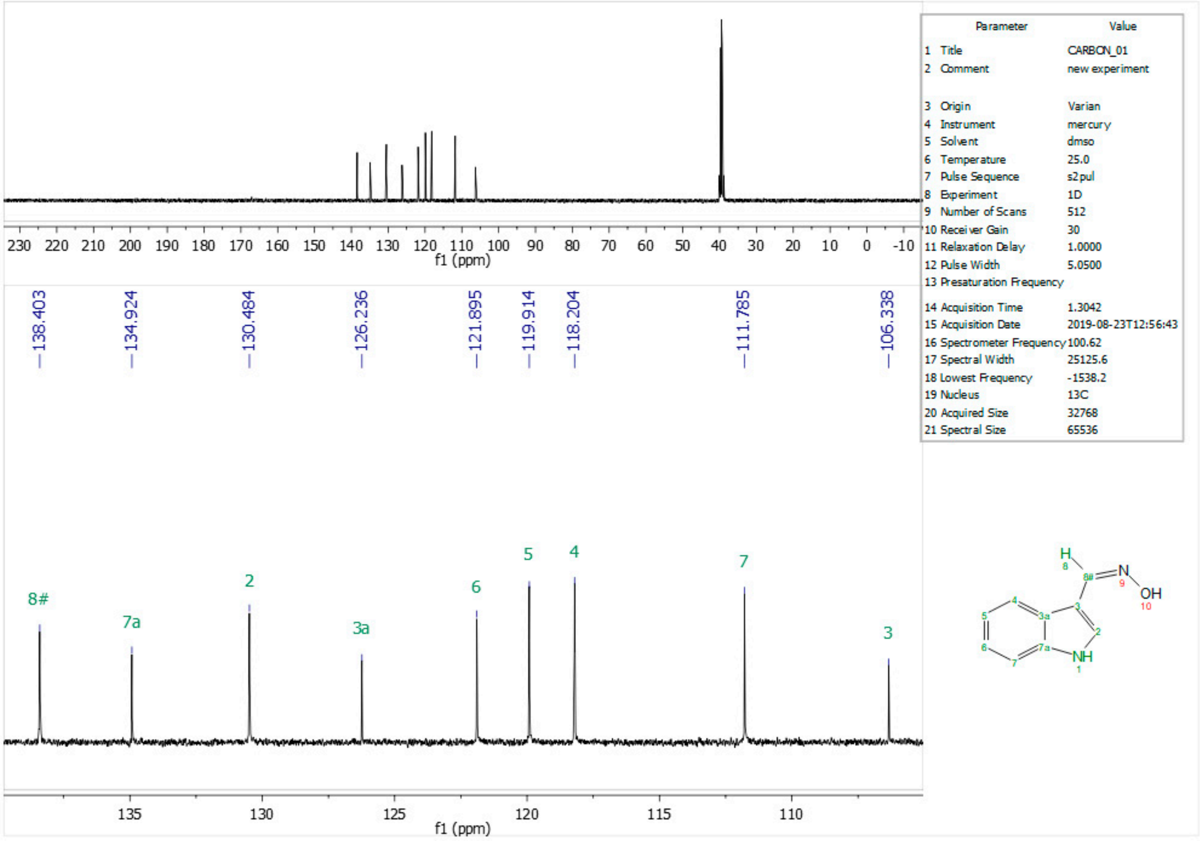

Fig. S29: ^{13}C NMR spectrum of *syn* indole-3-carboxaldehyde oxime 2a in DMSO.

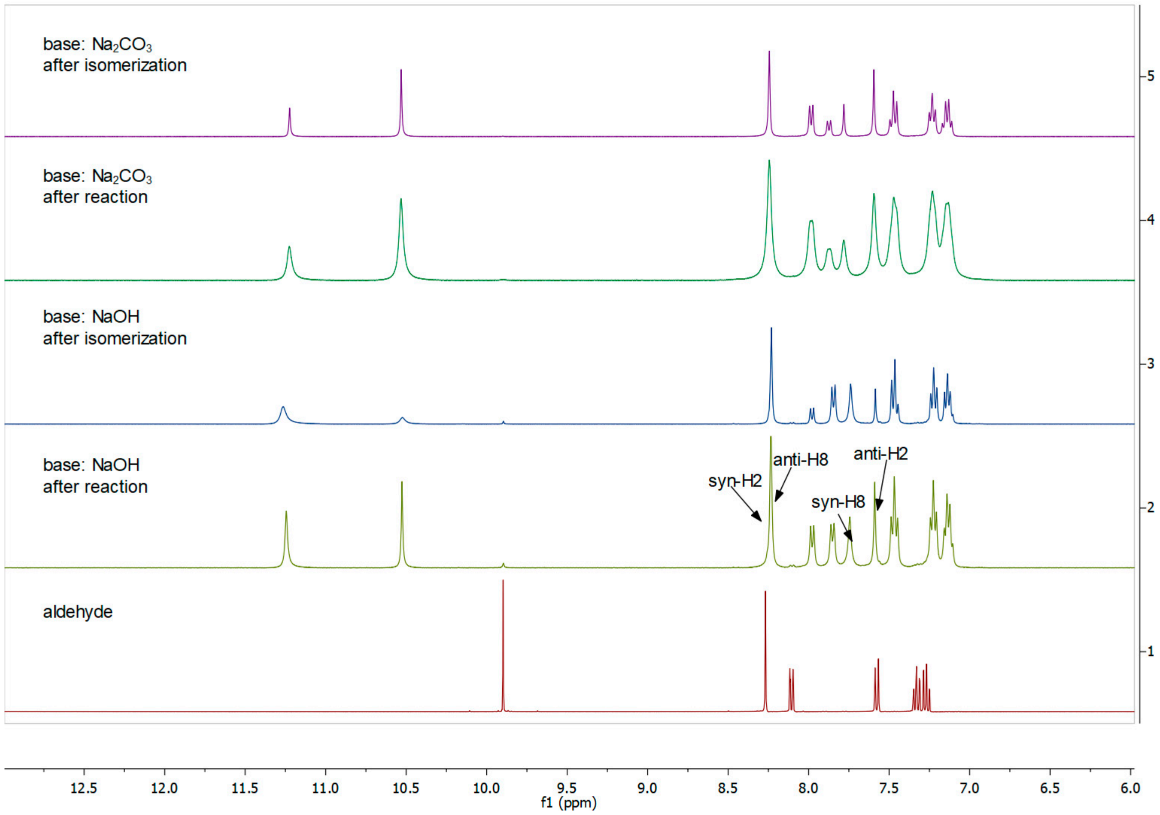

Fig. S30: NMR spectra of the products of the reaction of 1-methylindol-3-carboxaldehyde 3 with $NH_2OH.HCl$ and a base (indicated in figure) recorded after reaction and after isomerisation.

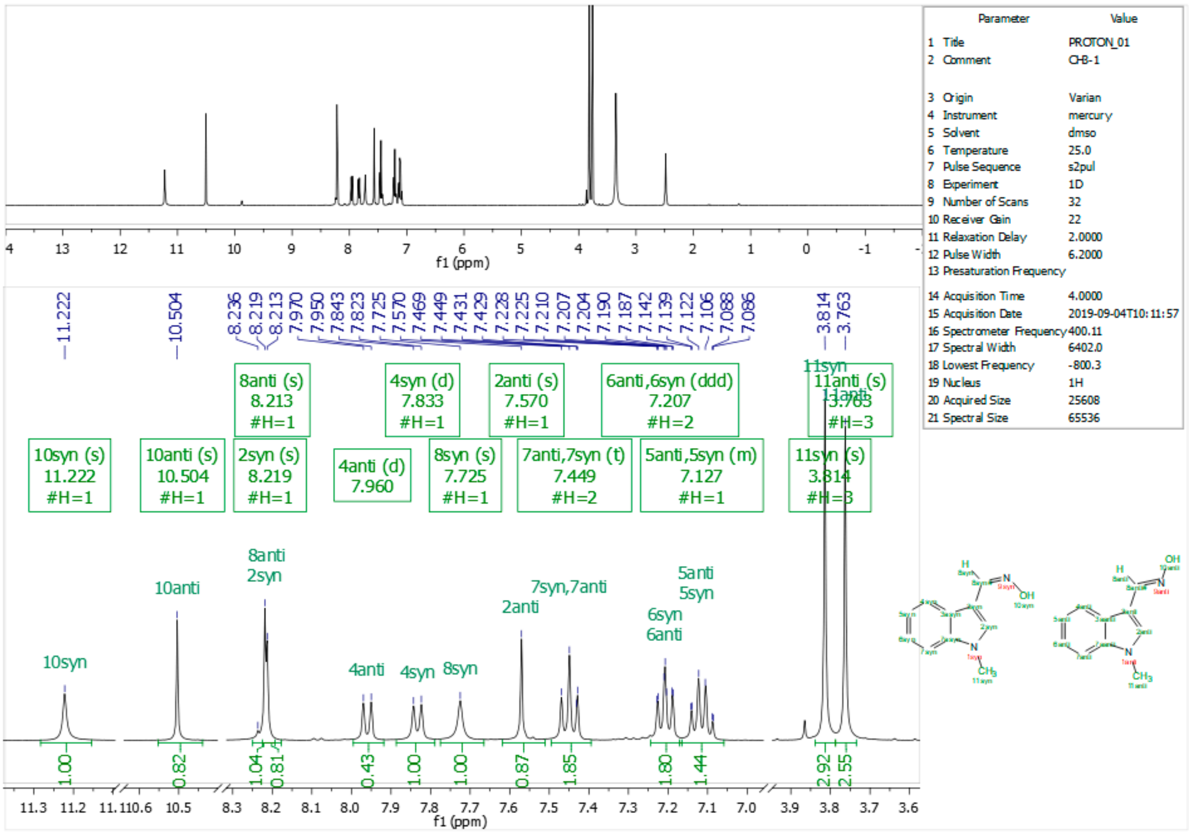

Fig. S31: ^{1}H NMR spectrum of mixture of *syn* 1-methylindole-3-carboxaldehyde oxime 3a and *anti* 1-methylindol-3-carboxaldehyde oxime 3a in DMSO.

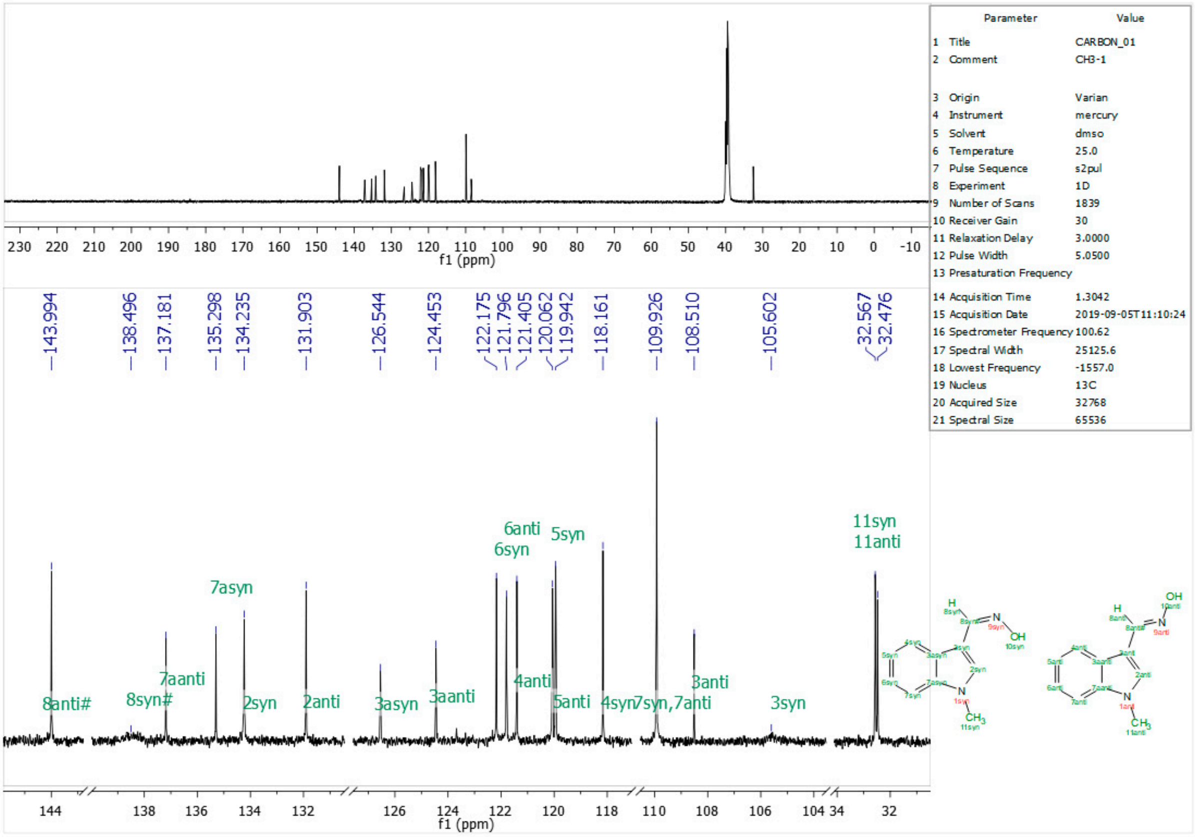

Fig. S32: ^{13}C NMR spectrum of mixture of *syn* 1-methylindole-3-carboxaldehyde oxime 3a and *anti* 1-methylindol-3-carboxaldehyde oxime 3a in DMSO.

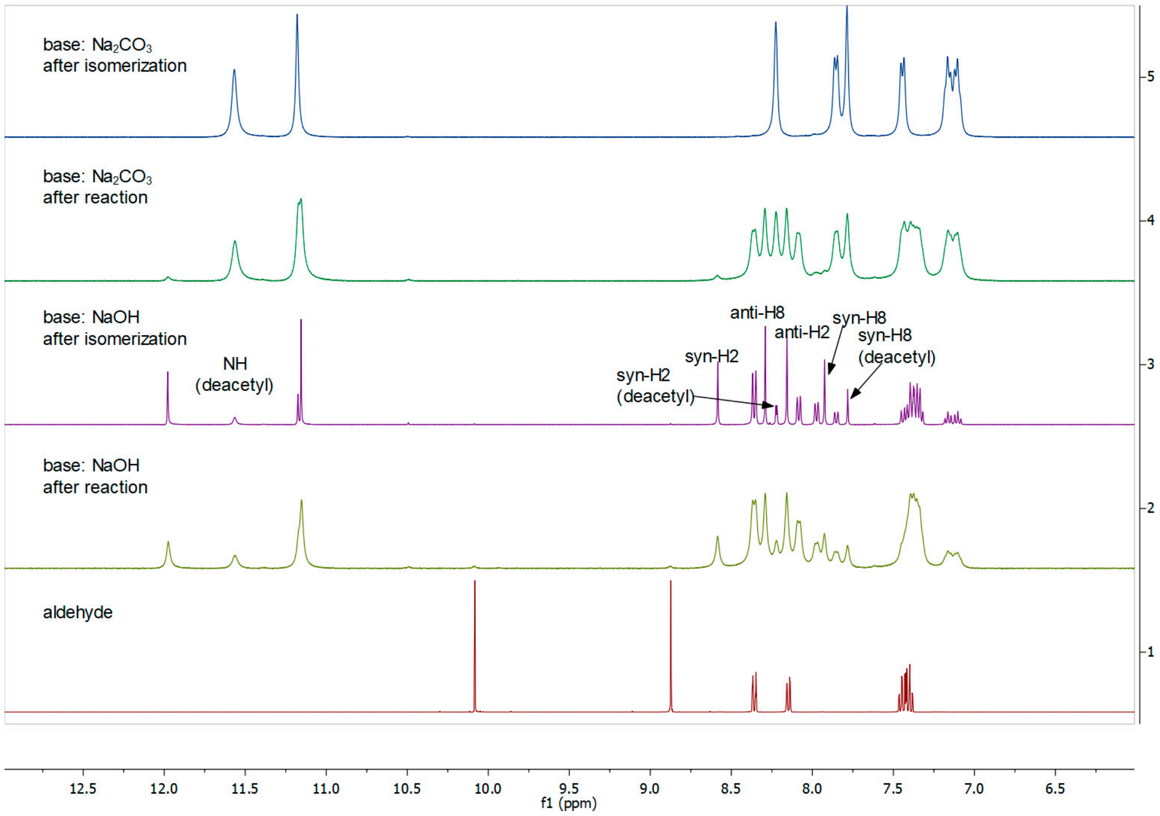

Fig. S33: NMR spectra of the products of the reaction of 1-acetylindol-3-carboxaldehyde 4 with $NH_2OH.HCl$ and a base (indicated in figure) recorded after reaction and after isomerisation.

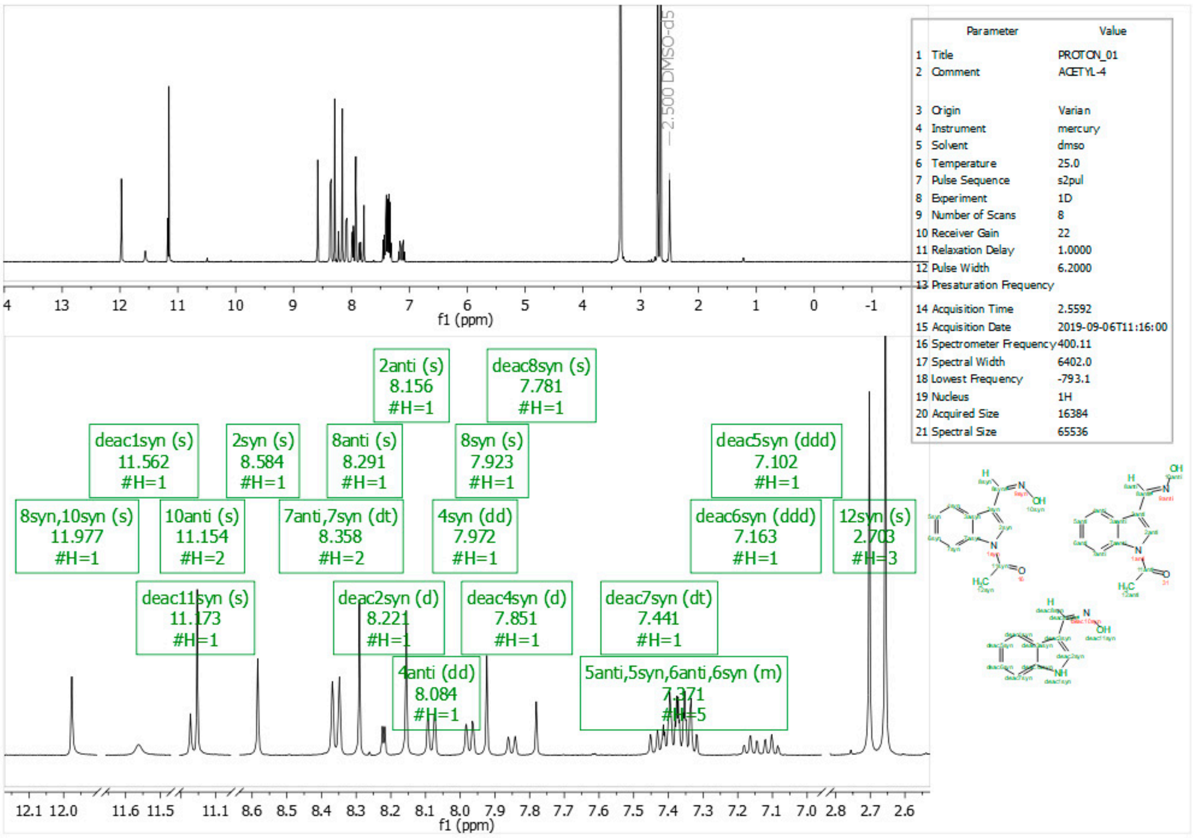

Fig. S34: ^{1}H NMR spectrum of mixture of *syn* 1-acetylindole-3-carboxaldehyde oxime 4a, *anti* 1-acetylindol-3-carboxaldehyde oxime 3a and deacetylated analog *syn* 2a in DMSO.

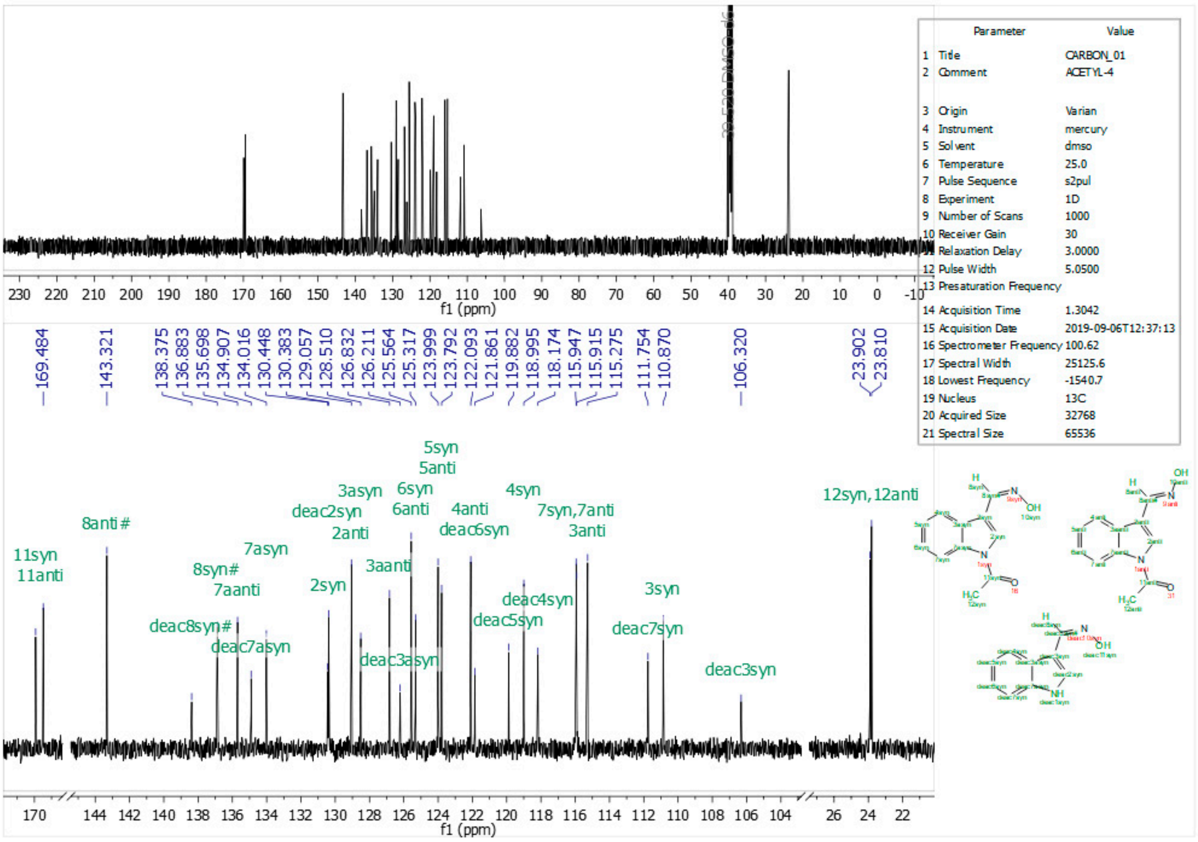

Fig. S35: ^{13}C NMR spectrum of mixture of *syn* 1-acetylindole-3-carboxaldehyde oxime 4a, *anti* 1-acetylindol-3-carboxaldehyde oxime 3a and deacetylated analog *syn* 2a in DMSO.

7. Description of characterization techniques

NMR spectra were recorded on a Mercury Plus NMR spectrometer (400.115 MHz for ^1H, 100.619 MHz, for ^{13}C) and a Varian VNMRS spectrometer (599.87 MHz for ^1H, 150.84 MHz for ^{13}C, and 60.79 MHz for ^{15}N) at 299.15 K. Chemical shifts (*d* in ppm) are referenced to solvent signals - DMSO-d_6 39.5 ppm and $CDCl_3$ 77.0 ppm for ^{13}C; DMSO-d_5 2.5 ppm and $CDCl_3$ 7.26 ppm for ^1H. External nitromethane (0.0 ppm) was used as a ^{15}N reference compound.

The XRD patterns were obtained using a D8 Advance diffractometer (Bruker Germany) with CuKα (40kV, 40 mA) radiation. All samples were scanned from 5° to 65° with steps 0.025° and 10 s counting time. The approximate crystallite sizes were calculated using the Scherrer's equation and using the Fityk software.

IR spectra were recorded using a Tensor 29 infrared spectrometer (Bruker, Germany) using the ATR method.

Melting points were determined on a Koffler micro melting point apparatus and are uncorrected.

The photographs of the crystals were obtained using a Nikon Eclipse Ci-S (Nikon, Japan) microscope equipped with a color CCD camera Progres C5 (Jenoptik, Germany).

For TEM studies, the samples were applied onto lacey carbon-coated Ni grids under dry conditions and without additional milling in order to prevent sample changes during the preparation procedure. The grids were additionally coated with a thin layer of carbon prior to TEM analyses to improve the surface conductivity. Transmission electron microscopy analyses were performed using a 200-kV microscope JEM 2100 (JEOL, Japan) with LaB_6 electron source and equipped with energy dispersive X-ray spectrometer (EDS) for chemical analyses. Due to the high sensitivity of the samples to high-energy electrons, a smaller condenser aperture and lower electron beam density were used for the observations and analyses.

8. References used in the Electronic Supplementary Information

1. Aakeroy, C. B.; Sinha, A. S.; Epa, K. N.; Spartz, C. L.; Desper, J., A versatile and green mechanochemical route for aldehyde-oxime conversions. *Chem. Commun.* **2012,** 48, (92), 11289-11291.
2. Sundberg, R. J., *The Chemistry of Indoles in Organic Chemistry*. Academic Press: New York, 1970; p 405.
3. Hanley, A. B.; Parsley, K. R.; Lewis, J. A.; Fenwick, G. R., Chemistry of Indole Glucosinolates - Intermediacy of Indol-3-Ylmethyl Isothiocyanates in the Enzymatic-Hydrolysis of Indole Glucosinolates. *J. Chem. Soc. Perkin. Trans. 1* **1990**, (8), 2273-2276.
4. Contreras, R. H.; Peralta, J. E., Angular dependence of spin-spin coupling constants. *Prog. Nucl. Magn. Reson. Spectrosc.* **2000,** 37, (4), 321-425.

5. Afonin, A. V.; Ushakov, I. A.; Vashchenko, A. V.; Simonenko, D. E.; Ivanov, A. V.; Vasil'tsov, A. M.; Mikhaleva, A. I.; Trofimov, B. A., C-H...N and C-H...O intramolecular hydrogen bonding effects in the ^{1}H, ^{13}C and ^{15}N NMR spectra of the configurational isomers of 1-vinylpyrrole-2-carbaldehyde oxime substantiated by DFT calculations. *Magn. Reson. Chem.* **2009,** 47, (2), 105-112.
6. Palm, A.; Werbin, H., The infrared spectra of alpha and beta oximes. *Can. J. Chem.* **1953,** 31, 1004-1008.
7. Kawasaki, T.; Somei, M., The first total syntheses of 9-methoxycarbazole-3-carboxaldehyde and methoxybrassinin. *Heterocycles* **1990,** 31, 1605-1607.
